# Supplementary material for: Drug repurposing for aging research using model organisms
Source: Aging Cell. 2017 Jun 16;16(5):1006–15. doi: 10.1111/acel.12626 (PMC5595691; doi:10.1111/acel.12626)
Supplement: Supplementary file 7 — Data S1 Zip‐Archive of all report cards. [file ACEL-16-1006-s007.zip › RC_ATP.pdf]

ATP

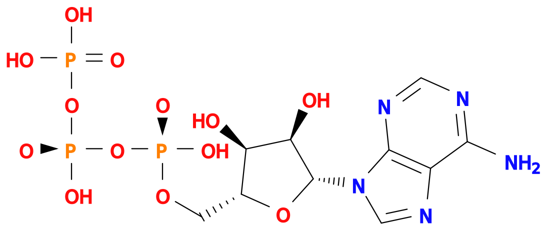

Database identifiers

ChEMBLCompound	CHEMBL14249
DrugBank	DB00171
CHEBI	15422
eMolecules	1934325

Ranking

	Rank	Score
Drosophila	284/697	0.583
C. elegans	573/591	0.0

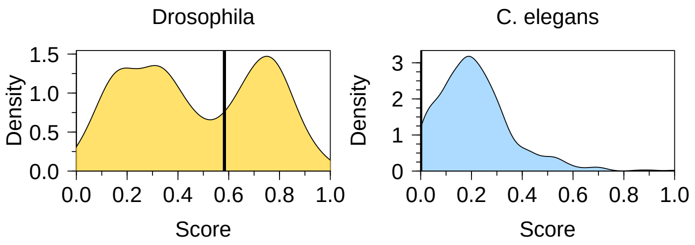

	Ageing implication	Domain conservation	Binding site conservation	Binding affinity	Bioavailability	Lipinski	Promiscuity	Purchasability	Drug approval	**Total**
Drosophila	1.0	0.977	1.0	0.862	(0.9)	-0.15	-0.2	0.1	0.075	0.583
C. elegans	1.0	0.976	1.0	0.862	0.206	-0.15	-0.2	0.1	0.075	0.0

Names

- **ATP**
- Adenosine triphosphate
- H4atp

Roles

nutraceutical, micronutrient, fundamental metabolite

Status

Approved drug (according to ChEMBL)	No
Classification (according to DrugBank)	approved, nutraceutical
Number of Rule of 5 violations	3
Binding affinity to original target in log units (RF-Score prediction)	6.83
Burns *C. elegans* bioavailability prediction	-6.93

Medical Information

Indicatation: For nutritional supplementation, also for treating dietary shortage or imbalance
Mechanism of action: ATP is able to store and transport chemical energy within cells. ATP also plays an important role in the synthesis of nucleic acids. ATP can be produced by various cellular processes, most typically in mitochondria by oxidative phosphorylation under the catalytic influence of ATP synthase. The total quantity of ATP in the human body is about 0.1 mole. The energy used by human cells requires the hydrolysis of 200 to 300 moles of ATP daily. This means that each ATP molecule is recycled 2000 to 3000 times during a single day. ATP cannot be stored, hence its consumption must closely follow its synthesis.
Toxicity: Oral LD50 in rats is > 2 g/kg.

(Information from DrugBank)

Compound Target Characteristics

3-phosphoinositide-dependent protein kinase 1

Best gene implication in ageing for this target family came from gene Q9Y1J3 via mapping the annotation from Ensembl WBGene00003965 via mapping the annotation from EntrezGene 180475 via mapping the annotation from GenAgeModels 0499 annotated in GenAge release 17.

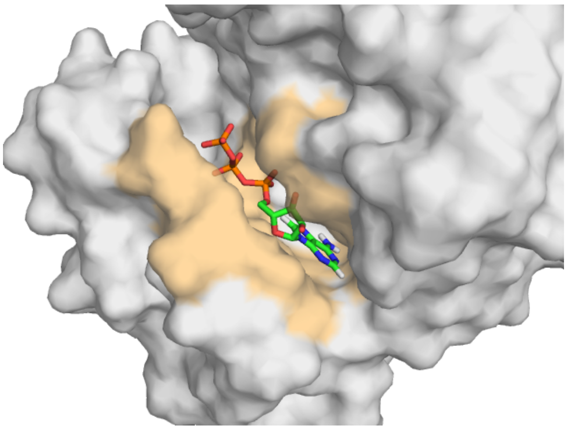

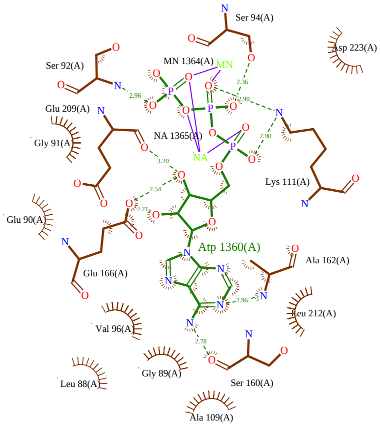

protein	amino acids contacts (binding site)
PDB:1h1w:chainA:O15530	L G E G S F S V A K V S Y A E E N L D
PDB:2biy:chainA:O15530	L G E G S F S V A K V S Y A E E N L D
PDB:3hrc:chainA:O15530	L G E G S F S V A K V S Y A E E N L D
PDB:3hrf:chainA:O15530	L G E G S F S V A K V S Y A E E N L D
PDB:4a06:chainA:O15530	L G E G S F S V A K V S Y A E E N L D
PDB:4a07:chainA:O15530	L G E G S F S V A K V S Y A E E N L D
PDB:4aw0:chainA:O15530	L G E G S F S V A K V S Y A E E N L D
PDB:4aw1:chainA:O15530	L G E G S F S V A K V S Y A E E N L D
PDB:4ct1:chainA:O15530	L G E G S F S V A K V S Y A E E N L D
PDB:4ct2:chainA:O15530	L G E G S F S V A K V S Y A E E N L D
tr:Q9UPJ8:Q9UPJ8_HUMAN	L G E G S F S V A K V S Y A E E N L D
tr:E9PER6:E9PER6_HUMAN	L G E G S F S V A K V S Y A E E N L D
tr:C9JWR9:C9JWR9_HUMAN	L G E G S F S V A K V S Y A E E N L D
sp:O15530:PDPK1_HUMAN	L G E G S F S V A K V S Y A E E N L D
sp:O55173:PDPK1_RAT	L G E G S F S V A K V S Y A E E N L D
tr:Q3UEW8:Q3UEW8_MOUSE	L G E G S F S V A K V S Y A E E N L D
tr:F2Z400:F2Z400_MOUSE	L G E G S F S V A K V S Y A E E N L D
tr:Q3TRL2:Q3TRL2_MOUSE	L G E G S F S V A K V S Y A E E N L D
tr:F2Z3X6:F2Z3X6_MOUSE	L G E G S F S V A K V S Y A E E N L D
sp:Q9Z2A0:PDPK1_MOUSE	L G E G S F S V A K V S Y A E E N L D
tr:Q810Z4:Q810Z4_MOUSE	L G E G S F S V A K V S Y A E E N L D
tr:A4V164:A4V164_DROME	I G E G S Y S V A K V T Y A D E N L D
tr:C7LAC8:C7LAC8_DROME	I G E G S Y S V A K V T Y A D E N L D
tr:H5V8B9:H5V8B9_DROME	I G E G S Y S V A K V T Y A D E N L D
sp:Q9W0V1:PDPK1_DROME	I G E G S Y S V A K V T Y A D E N L D
sp:Q9Y1J3:PDPK1_CAEEL	M G E G A Y S V A K T G L V D D N L D
sp:Q03407:PKH1_YEAST	L G D G S Y S V A K F E Y A D E N L D
sp:Q03306:PKH3_YEAST	L G H G S Y S V A K I D F A E E N L D

protein	whole protein ident	whole protein simil	domain-based ident	domain-based simil	contact-based ident	contact-based simil
PDB:1h1w:chainA:O15530	1.0	1.0	1.0	1.0	1.0	1.0
PDB:2biy:chainA:O15530	1.0	1.0	1.0	1.0	1.0	1.0
PDB:3hrc:chainA:O15530	0.99	1.0	0.99	1.0	1.0	1.0
PDB:3hrf:chainA:O15530	0.99	1.0	0.99	1.0	1.0	1.0
PDB:4a06:chainA:O15530	0.99	1.0	0.99	1.0	1.0	1.0
PDB:4a07:chainA:O15530	0.99	1.0	0.99	1.0	1.0	1.0
PDB:4aw0:chainA:O15530	0.99	0.99	0.99	0.99	1.0	1.0
PDB:4aw1:chainA:O15530	0.99	0.99	0.99	1.0	1.0	1.0
PDB:4ct1:chainA:O15530	0.96	0.99	0.96	0.99	1.0	1.0
PDB:4ct2:chainA:O15530	0.97	0.99	0.97	0.99	1.0	1.0
tr:Q9UPJ8:Q9UPJ8_HUMAN	0.66	0.66	1.0	1.0	1.0	1.0
tr:E9PER6:E9PER6_HUMAN	0.95	0.95	1.0	1.0	1.0	1.0
tr:C9JWR9:C9JWR9_HUMAN	0.95	0.96	0.91	0.91	1.0	1.0
sp:O15530:PDPK1_HUMAN	1.0	1.0	1.0	1.0	1.0	1.0
sp:O55173:PDPK1_RAT	0.95	0.98	0.99	1.0	1.0	1.0
tr:Q3UEW8:Q3UEW8_MOUSE	0.73	0.76	0.99	1.0	1.0	1.0
tr:F2Z400:F2Z400_MOUSE	0.75	0.79	0.99	1.0	1.0	1.0
tr:Q3TRL2:Q3TRL2_MOUSE	0.88	0.91	0.99	1.0	1.0	1.0
tr:F2Z3X6:F2Z3X6_MOUSE	0.9	0.93	0.99	1.0	1.0	1.0
sp:Q9Z2A0:PDPK1_MOUSE	0.95	0.98	0.99	1.0	1.0	1.0
tr:Q810Z4:Q810Z4_MOUSE	0.93	0.97	0.99	1.0	1.0	1.0
tr:A4V164:A4V164_DROME	0.31	0.56	0.43	0.68	0.79	0.93
tr:C7LAC8:C7LAC8_DROME	0.28	0.5	0.43	0.68	0.79	0.93
tr:H5V8B9:H5V8B9_DROME	0.28	0.5	0.43	0.68	0.79	0.93
sp:Q9W0V1:PDPK1_DROME	0.28	0.5	0.43	0.68	0.79	0.93
sp:Q9Y1J3:PDPK1_CAEEL	0.28	0.61	0.35	0.7	0.53	0.77
sp:Q03407:PKH1_YEAST	0.21	0.48	0.46	0.79	0.74	0.87
sp:Q03306:PKH3_YEAST	0.15	0.35	0.4	0.69	0.74	0.91

Pdk1 (FBgn0020386) associated phenotypes

cell autonomous, chemical conditional, chemical resistant, chemical sensitive, decreased cell size,

developmental rate defective, increased cell death, increased cell size, male limited, neuroanatomy defective, nutrition conditional, partially lethal - majority die, small body, somatic clone

(Information from FlyBase)

Pdk1 (UniProt:Q9W0V1) annotation

Function: Serine/threonine kinase required for embryonic development. Inhibits apoptosis. Acts in the insulin receptor transduction pathway which regulates cell growth and organ size, by phosphorylating and activating Akt1 and S6k. May be involved in axonal pathfinding and synaptogenesis, and in spermatogenesis. (PubMed:10336630, PubMed:11301252, PubMed:11344272, PubMed:11752451, PubMed:11862217, PubMed:15238523, PubMed:9368760).

Subcellular location: Cytoplasm

Developmental stage: Highly expressed in Malpighian tubule primordia from stage late 11 to 13. Expressed in hindgut at stage 13. After stage 14, ubiquitously expressed at low levels. (PubMed:11301252).

Domain: The PIF-pocket is a small lobe in the catalytic domain required by the enzyme for the binding to the hydrophobic motif of its substrates. It is an allosteric regulatory site that can accommodate small compounds acting as allosteric inhibitors. (UniProtKB:O15530).

Disruption phenotype: Death during the second instar stage. (PubMed:11752451).

(Information from UniProt)

pdk-1 (WBGene00003965) associated phenotypes

DMPP resistant, carbon dioxide avoidance variant, dauer constitutive, dauer recovery inhibited, egg laying variant, extended life span, long, reduced brood size, shortened life span, social feeding increased

(Information from WormBase)

pdk-1 (UniProt:Q9Y1J3) annotation

Function: Involved in the daf-2/insulin receptor-like transduction pathway, which controls longevity and prevents developmental arrest at the dauer stage. Phosphorylates and activates sgk-1, akt-1 and akt-2. (PubMed:10364160, PubMed:15068796).

Subunit: Interacts directly with sgk-1, akt-1 and akt-2.(PubMed:15068796).

Subcellular location: Cytoplasm

Developmental stage: Expressed in late stage embryos and throughout life. At L1, expressed in neurons, intestinal cells and hypodermal cells. In adults, expressed in the somatic gonad. (PubMed:10364160).

Domain: The PIF-pocket is a small lobe in the catalytic domain required by the enzyme for the binding to the hydrophobic motif of its substrates. It is an allosteric regulatory site that can accommodate small compounds acting as allosteric inhibitors. (UniProtKB:O15530).

(Information from UniProt)

ATP-dependent molecular chaperone HSP82

Best gene implication in ageing for this target family came from gene Q18688 annotated in UniProt release 2014_02. Annotation GO 8340 (determination of adult lifespan) was Inferred from Genetic Interaction

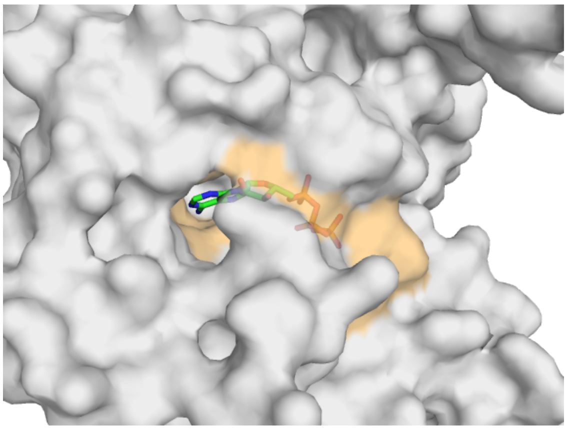 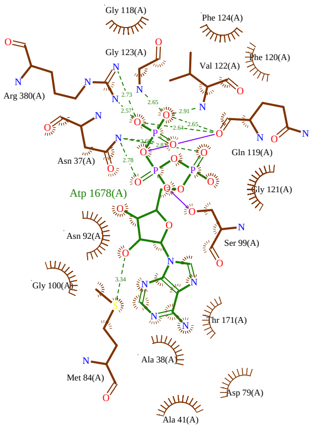

protein	amino acids contacts (binding site)																	
PDB:2cg9:chainA:P02829	E	N	A	A	D	M	N	S	G	G	Q	F	G	V	G	F	T	R
tr:K9JA46:K9JA46_HUMAN	E	N	S	A	D	M	N	S	G	G	Q	F	G	V	G	F	T	R
sp:P07900:HS90A_HUMAN	E	N	S	A	D	M	N	S	G	G	Q	F	G	V	G	F	T	R
sp:P82995:HS90A_RAT	E	N	S	A	D	M	N	S	G	G	Q	F	G	V	G	F	T	R
tr:Q3TKB9:Q3TKB9_MOUSE	E	N	S	A	D	M	N	S	G	G	Q	F	G	V	G	F	T	R
tr:Q3TKG0:Q3TKG0_MOUSE	E	N	S	A	D	M	N	S	G	G	Q	F	G	V	G	F	T	R
sp:P07901:HS90A_MOUSE	E	N	S	A	D	M	N	S	G	G	Q	F	G	V	G	F	T	R
tr:Q80Y52:Q80Y52_MOUSE	E	N	S	A	D	M	N	S	G	G	Q	F	G	V	G	F	T	R
sp:P02828:HSP83_DROME	E	N	A	A	D	M	N	S	G	G	Q	F	G	V	G	F	T	R
sp:Q18688:HSP90_CAEEL	E	N	A	A	D	M	N	S	G	G	Q	F	G	V	G	F	T	R
sp:P02829:HSP82_YEAST	E	N	A	A	D	M	N	S	G	G	Q	F	G	V	G	F	T	R

protein	whole protein ident	whole protein simil	domain-based ident	domain-based simil	contact-based ident	contact-based simil
PDB:2cg9:chainA:P02829	1.0	1.0	1.0	1.0	1.0	1.0
tr:K9JA46:K9JA46_HUMAN	0.6	0.85	0.68	0.9	0.94	0.95
sp:P07900:HS90A_HUMAN	0.6	0.85	0.68	0.9	0.94	0.95
sp:P82995:HS90A_RAT	0.59	0.85	0.68	0.9	0.94	0.95
tr:Q3TKB9:Q3TKB9_MOUSE	0.48	0.65	0.68	0.9	0.94	0.95
tr:Q3TKG0:Q3TKG0_MOUSE	0.48	0.65	0.68	0.9	0.94	0.95
sp:P07901:HS90A_MOUSE	0.59	0.85	0.68	0.9	0.94	0.95
tr:Q80Y52:Q80Y52_MOUSE	0.59	0.85	0.68	0.9	0.94	0.95
sp:P02828:HSP83_DROME	0.6	0.86	0.66	0.91	1.0	1.0
sp:Q18688:HSP90_CAEEL	0.6	0.85	0.66	0.9	1.0	1.0
sp:P02829:HSP82_YEAST	1.0	1.0	1.0	1.0	1.0	1.0

Hsp83 (FBgn0001233) associated phenotypes

cell cycle defective, cell polarity defective, developmental rate defective, female semi-fertile, lethal - all die before end of pupal stage, lethal - all die during third instar larval stage, partially lethal, partially lethal - majority die, short lived

(Information from FlyBase)

Hsp83 (UniProt:P02828) annotation

Function: Molecular chaperone that promotes the maturation, structural maintenance and proper regulation of specific target proteins involved for instance in cell cycle control and signal transduction. Undergoes a functional cycle that is linked to its ATPase activity. This cycle probably induces conformational changes in the client proteins, thereby causing their activation. Interacts dynamically with various co-chaperones that modulate its substrate recognition, ATPase cycle and chaperone function. Together with Hop and piwi, mediates canalization, also known as developmental robustness, likely via epigenetic silencing of existing genetic variants and suppression of transposon-induced new genetic variation. Required for piRNA biogenesis by facilitating loading of piRNAs into PIWI proteins. (PubMed:21186352, PubMed:22902557).

Subunit: Homodimer (By similarity). Forms a complex with Hop and piwi; probably Hop mediates

the interaction between piwi and Hsp83. Interacts with shu. (, PubMed:21186352, PubMed:22902557).
Subcellular location: Cytoplasm.
Induction: In contrast to other major heat shock proteins, this one is also expressed at normal growth temperatures. It is also developmentally expressed during oogenesis.
Domain: The TPR repeat-binding motif mediates interaction with TPR repeat-containing proteins.

(Information from UniProt)

daf-21 (WBGene00000915) associated phenotypes
chemotaxis variant, dauer constitutive, dauer recovery variant, larval lethal, lethal, odorant chemosensory response variant, sluggish, sterile

(Information from WormBase)

daf-21 (UniProt:Q18688) annotation
Function: Molecular chaperone that promotes the maturation, structural maintenance and proper regulation of specific target proteins involved for instance in cell cycle control and signal transduction. Undergoes a functional cycle that is linked to its ATPase activity. This cycle probably induces conformational changes in the client proteins, thereby causing their activation. Interacts dynamically with various co-chaperones that modulate its substrate recognition, ATPase cycle and chaperone function. By stabilizing the receptor-type guanylate cyclase daf-11 or another signal transduction component that regulates cGMP levels, plays a role in dauer formation and chemotaxis to non-volatile and volatile attractants detected by AWC sensory neurons (PubMed:10790386, PubMed:7828815). Participates in the control of cell cycle progression at the prophase/metaphase transition in oocyte development by ensuring the activity of wee-1.3 kinase, which negatively regulates cdk-1 through its phosphorylation (PubMed:16466390). Regulates yap-1 nuclear export after heat shock treatment (PubMed:23396260). (PubMed:10790386, PubMed:16466390, PubMed:23396260, PubMed:7828815).
Subunit: Homodimer. (UniProtKB:P08238).
Subcellular location: Cytoplasm, perinuclear region (PubMed:12950278). Note=Perinuclear region of somatic cells.
Tissue specificity: In the embryo comma stage, expression is strongly detected in cells of the head region and less so in other areas. In early larvae, expressed in postembryonic germ cells derived from Z2 and Z3 cells and the head region, in both hermaphrodites and males. Under heat stress conditions, larval expression is not only detected in germ cells, but also all over the body. In adult hermaphrodites, expression is localized uniquely in the germ cells. (PubMed:12950278).
Developmental stage: Highly expressed throughout development. (PubMed:10790386).
Domain: The TPR repeat-binding motif mediates interaction with TPR repeat-containing proteins.

(Information from UniProt)

Insulin receptor

Best gene implication in ageing for this target family came from gene P09208 annotated in UniProt release 2014_02. Annotation GO 8340 (determination of adult lifespan) was Inferred from Mutant Phenotype

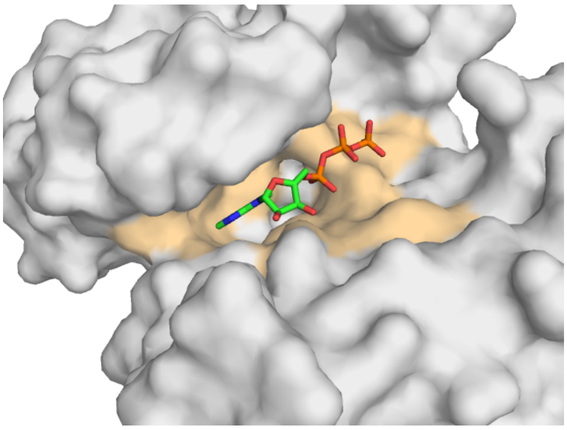

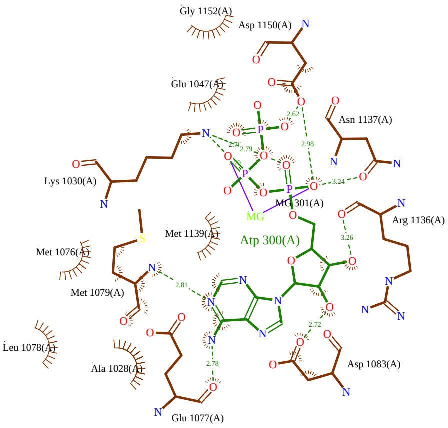
Gly 1152(A)
Asp 1150(A)
Glu 1047(A)
Asn 1137(A)
Lys 1030(A)
MG 301(A)
MG
Arg 1136(A)
Met 1139(A)
Atp 300(A)
Met 1076(A)
Met 1079(A)
Leu 1078(A)
Ala 1028(A)
Asp 1083(A)
Glu 1077(A)
2.62
2.98
2.79
3.24
3.26
2.81
2.72
2.78

protein	amino acids contacts (binding site)
PDB:3bu5:chainA:P06213	A K E M E L M D R N M D G
sp:P08069:IGF1R_HUMAN	A K E M E L M D R N M D G
tr:C9J5X1:C9J5X1_HUMAN	A K E M E L M D R N M D G
sp:P06213:INSR_HUMAN	A K E M E L M D R N M D G
tr:F1LN53:F1LN53_RAT	A K E M E L M D R N M D G
tr:F1LPL6:F1LPL6_RAT	A K E M E L M D R N M D G
tr:Q3UVJ3:Q3UVJ3_MOUSE	A K E M E L M D R N M D G
tr:E9QNX9:E9QNX9_MOUSE	A K E M E L M D R N M D G
sp:P15208:INSR_MOUSE	A K E M E L M D R N M D G
sp:Q60751:IGF1R_MOUSE	A K E M E L M D R N M D G
tr:M4TKR7:M4TKR7_MOUSE	A K E M E L M D R N M D G
tr:G7H807:G7H807_DROME	A K E M E L M D R N M D G
tr:D0UY96:D0UY96_DROME	A K E M E L M D R N M D G
tr:D0UY86:D0UY86_DROME	A K E M E L M D R N M D G
tr:D0UY84:D0UY84_DROME	A K E M E L M D R N M D G
tr:D0UY85:D0UY85_DROME	A K E M E L M D R N M D G
tr:D0UY80:D0UY80_DROME	A K E M E L M D R N M D G
tr:D0UY81:D0UY81_DROME	A K E M E L M D R N M D G
tr:D0UY88:D0UY88_DROME	A K E M E L M D R N M D G
tr:D0UY89:D0UY89_DROME	A K E M E L M D R N M D G
tr:D0UYA1:D0UYA1_DROME	A K E M E L M D R N M D G
tr:D0UYA0:D0UYA0_DROME	A K E M E L M D R N M D G
tr:D0UYA6:D0UYA6_DROME	A K E M E L M D R N M D G
tr:D0UY91:D0UY91_DROME	A K E M E L M D R N M D G
tr:D0UY97:D0UY97_DROME	A K E M E L M D R N M D G
tr:D0UY78:D0UY78_DROME	A K E M E L M D R N M D G
tr:D0UY95:D0UY95_DROME	A K E M E L M D R N M D G
tr:D0UY94:D0UY94_DROME	A K E M E L M D R N M D G
tr:D0UY75:D0UY75_DROME	A K E M E L M D R N M D G
tr:D0UY77:D0UY77_DROME	A K E M E L M D R N M D G
tr:D0UY70:D0UY70_DROME	A K E M E L M D R N M D G
tr:D0UYA5:D0UYA5_DROME	A K E M E L M D R N M D G
tr:D0UYA9:D0UYA9_DROME	A K E M E L M D R N M D G
tr:D0UY99:D0UY99_DROME	A K E M E L M D R N M D G
tr:D0UYA4:D0UYA4_DROME	A K E M E L M D R N M D G
tr:D0UYA8:D0UYA8_DROME	A K E M E L M D R N M D G
tr:D0UY98:D0UY98_DROME	A K E M E L M D R N M D G
tr:D0UYA7:D0UYA7_DROME	A K E M E L M D R N M D G
tr:C7C412:C7C412_DROME	A K E M E L M D R N M D G
tr:C7C413:C7C413_DROME	A K E M E L M D R N M D G
tr:C7C410:C7C410_DROME	A K E M E L M D R N M D G
tr:C7C411:C7C411_DROME	A K E M E L M D R N M D G
tr:C7C414:C7C414_DROME	A K E M E L M D R N M D G
tr:C7C415:C7C415_DROME	A K E M E L M D R N M D G
tr:C7C417:C7C417_DROME	A K E M E L M D R N M D G
sp:P09208:INSR_DROME	A K E M E L M D R N M D G
tr:C7C416:C7C416_DROME	A K E M E L M D R N M D G
sp:Q968Y9:INSR_CAEEL	A K E M E M M N R N M D G

	whole protein		domain-based		contact-based	
protein	ident	simil	ident	simil	ident	simil
PDB:3bu5:chainA:P06213	1.0	1.0	1.0	1.0	1.0	1.0
sp:P08069:IGF1R_HUMAN	0.55	0.83	0.83	0.95	1.0	1.0
tr:C9J5X1:C9J5X1_HUMAN	0.55	0.83	0.83	0.95	1.0	1.0
sp:P06213:INSR_HUMAN	1.0	1.0	1.0	1.0	1.0	1.0
tr:F1LN53:F1LN53_RAT	0.85	0.88	0.97	0.99	1.0	1.0
tr:F1LPL6:F1LPL6_RAT	0.86	0.89	0.97	0.99	1.0	1.0
tr:Q3UVJ3:Q3UVJ3_MOUSE	0.37	0.56	0.82	0.95	1.0	1.0
tr:E9QNX9:E9QNX9_MOUSE	0.55	0.83	0.82	0.95	1.0	1.0
sp:P15208:INSR_MOUSE	0.94	0.98	0.97	0.99	1.0	1.0
sp:Q60751:IGF1R_MOUSE	0.55	0.83	0.82	0.95	1.0	1.0
tr:M4TKR7:M4TKR7_MOUSE	0.95	0.98	0.97	0.99	1.0	1.0
tr:G7H807:G7H807_DROME	0.12	0.21	0.63	0.85	1.0	1.0
tr:D0UY96:D0UY96_DROME	0.22	0.47	0.63	0.85	1.0	1.0
tr:D0UY86:D0UY86_DROME	0.22	0.47	0.63	0.85	1.0	1.0
tr:D0UY84:D0UY84_DROME	0.22	0.47	0.63	0.85	1.0	1.0
tr:D0UY85:D0UY85_DROME	0.22	0.47	0.63	0.85	1.0	1.0
tr:D0UY80:D0UY80_DROME	0.22	0.47	0.63	0.85	1.0	1.0
tr:D0UY81:D0UY81_DROME	0.22	0.47	0.63	0.85	1.0	1.0
tr:D0UY88:D0UY88_DROME	0.22	0.47	0.63	0.85	1.0	1.0
tr:D0UY89:D0UY89_DROME	0.22	0.47	0.63	0.85	1.0	1.0
tr:D0UYA1:D0UYA1_DROME	0.22	0.47	0.63	0.85	1.0	1.0
tr:D0UYA0:D0UYA0_DROME	0.22	0.47	0.63	0.85	1.0	1.0
tr:D0UYA6:D0UYA6_DROME	0.22	0.47	0.63	0.85	1.0	1.0
tr:D0UY91:D0UY91_DROME	0.22	0.47	0.63	0.85	1.0	1.0
tr:D0UY97:D0UY97_DROME	0.22	0.47	0.63	0.85	1.0	1.0
tr:D0UY78:D0UY78_DROME	0.22	0.47	0.63	0.85	1.0	1.0
tr:D0UY95:D0UY95_DROME	0.22	0.47	0.63	0.85	1.0	1.0
tr:D0UY94:D0UY94_DROME	0.22	0.47	0.63	0.85	1.0	1.0
tr:D0UY75:D0UY75_DROME	0.22	0.47	0.63	0.85	1.0	1.0
tr:D0UY77:D0UY77_DROME	0.22	0.47	0.63	0.85	1.0	1.0
tr:D0UY70:D0UY70_DROME	0.22	0.47	0.63	0.85	1.0	1.0
tr:D0UYA5:D0UYA5_DROME	0.22	0.47	0.63	0.85	1.0	1.0
tr:D0UYA9:D0UYA9_DROME	0.22	0.47	0.63	0.85	1.0	1.0
tr:D0UY99:D0UY99_DROME	0.22	0.47	0.63	0.85	1.0	1.0
tr:D0UYA4:D0UYA4_DROME	0.22	0.47	0.63	0.85	1.0	1.0
tr:D0UYA8:D0UYA8_DROME	0.22	0.47	0.63	0.85	1.0	1.0
tr:D0UY98:D0UY98_DROME	0.22	0.47	0.63	0.85	1.0	1.0
tr:D0UYA7:D0UYA7_DROME	0.22	0.46	0.63	0.85	1.0	1.0
tr:C7C412:C7C412_DROME	0.21	0.46	0.63	0.85	1.0	1.0
tr:C7C413:C7C413_DROME	0.21	0.46	0.63	0.85	1.0	1.0
tr:C7C410:C7C410_DROME	0.21	0.46	0.63	0.85	1.0	1.0
tr:C7C411:C7C411_DROME	0.21	0.46	0.63	0.85	1.0	1.0
tr:C7C414:C7C414_DROME	0.21	0.46	0.63	0.85	1.0	1.0
tr:C7C415:C7C415_DROME	0.21	0.46	0.63	0.85	1.0	1.0
tr:C7C417:C7C417_DROME	0.21	0.46	0.63	0.85	1.0	1.0
sp:P09208:INSR_DROME	0.21	0.46	0.63	0.85	1.0	1.0
tr:C7C416:C7C416_DROME	0.21	0.46	0.63	0.85	1.0	1.0
sp:Q968Y9:INSR_CAEEL	0.21	0.51	0.45	0.78	0.85	0.97

InR (FBgn0013984) associated phenotypes

RU486 conditional, aging defective, body size defective, cell autonomous, cell number defective, cell size defective, chemical sensitive, circadian rhythm defective, conditional, decreased cell number, decreased cell size, developmental rate defective, dominant, feeding behavior defective, heat sensitive, hyperplasia, increased cell size, lethal - all die before end of second instar larval stage, long lived, mating defective, melanotic mass phenotype, neuroanatomy defective, neurophysiology defective, oxidative stress response defective, partially, partially lethal, partially lethal - majority die, short lived, size defective, sleep defective, small body, somatic clone, some die during pupal stage, some die during second instar larval stage, starvation stress response defective, stress response defective

(Information from FlyBase)

InR (UniProt:P09208) annotation

Function: Has a ligand-stimulated tyrosine-protein kinase activity. Required for cell survival. Reg-

ulates body size and organ size by altering cell number and cell size in a cell- autonomous manner. Involved in the development of the embryonic nervous system, and is necessary for axon guidance and targeting in the visual system. Also plays a role in life-span determination. (PubMed:10455177, PubMed:11250149, PubMed:11292875, PubMed:12702880, PubMed:7628438, PubMed:8603594).
Cofactor: Mn(2+)
Enzyme regulation: Autophosphorylation activates the kinase activity.
Subunit: Tetramer of 2 alpha and 2 beta chains linked by disulfide bonds. The alpha chains contribute to the formation of the ligand- binding domain, while the beta chains carry the kinase domain. When autophosphorylated, the beta-subunit binds the SH2 and SH3 domains of the adapter protein Dock. The beta subunit also binds and tyrosine phosphorylates the insulin receptor substrate Chico.
Subcellular location: Membrane; Single-pass type I membrane protein.
Tissue specificity: Widely distributed throughout the embryo. Expressed at high levels in the embryonic nervous system. Larval expression is limited to the nervous system and all imaginal disks. Expressed at high levels in the adult nervous system and ovaries. (PubMed:2454394, PubMed:7628438).
Developmental stage: Expressed throughout development. Embryonic expression is most prominent 8 to 12 hours after egg laying. (PubMed:2454394, PubMed:3014506).
Ptm: The 280 kDa proreceptor is proteolytically processed to form a 120 kDa alpha subunit and a 170 kDa beta subunit. The beta subunit undergoes cell-specific cleavage to generate a 90 kDa beta subunit and a free 60 kDa C-terminal subunit. Both the 90 kDa and the 170 kDa beta subunits can assemble with the alpha subunits to form mature receptors. (PubMed:7628438).
Ptm: Autophosphorylated on tyrosine residues in response to exogenous insulin. (PubMed:18327897).
Ptm: Phosphorylation of Tyr-1354 is required for Chico-binding.

(Information from UniProt)

daf-2 (WBGene00000898) associated phenotypes
DMPP resistant, L2 larval development variant, aging variant, anoxia resistant, autophagy variant, brood size variant, carbohydrate metabolism variant, carbon dioxide avoidance variant, dauer arrest variant, dauer constitutive, dauer formation variant, diet induced life span variant, drug response variant, egg laying variant, extended life span, fat content increased, gene expression level high, gene expression variant, hormetic temperature induced life span extension defective, larval lethal, lethal, lipid composition variant, lipid metabolism variant, lipid synthesis increased, mRNA expression variant, mRNA levels increased, mate searching variant, mianserin resistant, no dauer recovery, organ senescence variant, organism UV resistant, organism dessication response variant, organism hypersensitive dessication, organism oxidative stress response variant, organism physiology variant, organism starvation response variant, pathogen resistance increased, protein expression increased, protein phosphorylation increased, protein phosphorylation reduced, quiescence variant, reduced brood size, reproductive longevity extended, response to food variant, roaming reduced, spontaneous spicule protraction, sterile, temperature influenced life span variant, thermotolerance increased, transgene expression increased, transgene expression reduced, transgene subcellular localization variant, yolk synthesis variant

(Information from WormBase)

daf-2 (UniProt:Q968Y9) annotation
Function: An insulin receptor-like protein which regulates metabolism, controls longevity and prevents developmental arrest at the dauer stage. Binding of INS family members may either stimulate, or antagonize, association of the receptor with downstream mediators such as pdk-1 and age-1. Required for the response to environmental stimuli such as food, pheromone, and temperature. Role in immune function and pathogen resistance. (PubMed:11274053, PubMed:18782349, PubMed:9252323, PubMed:9790527).
Cofactor: Mn(2+) Evidence=(UniProtKB:P06213);

Enzyme regulation: Autophosphorylation activates the kinase activity (By similarity). Interaction with shc-1 may inhibits its activity (PubMed:18832074). (UniProtKB:P06213, PubMed:18832074).
Subunit: Tetramer of 2 alpha and 2 beta chains linked by disulfide bonds. The alpha chains contribute to the formation of the ligand- binding domain, while the beta chains carry the kinase domain (By similarity). Interacts (via cytoplasmic domain) with shc-1 (PID domain) (PubMed:18832074). (UniProtKB:P06213, PubMed:18832074).
Subcellular location: Membrane (UniProtKB:P06213); Single-pass type I membrane protein (UniProtKB:P06213).
Disruption phenotype: Accumulation of fat, pigmented intestine, increased life span, increased dauer formation and increased resistance to pathogens. Severe loss of function mutants display recessive early embryonic lethality. (PubMed:11274053, PubMed:18245374, PubMed:18782349, PubMed:9252323, PubMed:9790527).

(Information from UniProt)

Sodium/potassium-transporting ATPase subunit alpha-1

Best gene implication in ageing for this target family came from gene P13607 annotated in UniProt release 2014_02. Annotation GO 8340 (determination of adult lifespan) was Inferred from Mutant Phenotype

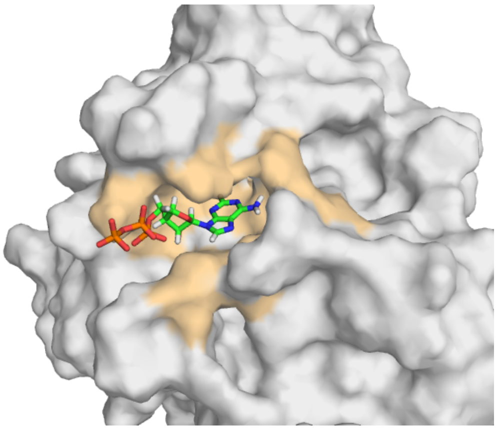

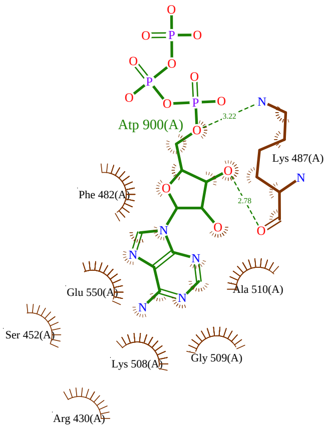

protein	amino acids contacts (binding site)							
PDB:1mo8:chainA:P06685	R	S	F	K	K	G	A	E
tr:M0R116:M0R116_HUMAN	R	S	F	K	K	G	A	E
tr:B1AKY9:B1AKY9_HUMAN	R	S	F	K	K	G	A	E
sp:P13637:AT1A3_HUMAN	R	S	F	K	K	G	A	E
sp:P05023:AT1A1_HUMAN	R	S	F	K	K	G	A	E
sp:P50993:AT1A2_HUMAN	R	S	F	K	K	G	A	E
sp:Q13733:AT1A4_HUMAN	R	S	F	K	K	G	A	E
sp:P54707:AT12A_HUMAN	R	S	F	K	K	G	A	E
sp:P20648:ATP4A_HUMAN	R	S	F	K	K	G	A	E
sp:P06687:AT1A3_RAT	R	S	F	K	K	G	A	E
tr:Q5PQV3:Q5PQV3_RAT	R	S	F	K	K	G	A	E
sp:Q64541:AT1A4_RAT	R	S	F	K	K	G	A	E
sp:P06686:AT1A2_RAT	R	S	F	K	K	G	A	E
sp:P06685:AT1A1_RAT	R	S	F	K	K	G	A	E
tr:F1LRK1:F1LRK1_RAT	R	S	F	K	K	G	A	E
tr:G3V8S4:G3V8S4_RAT	R	S	F	K	K	G	A	E
tr:D3YVX6:D3YVX6_MOUSE	R	S	F	K	K	G	A	E
tr:Q8R0E8:Q8R0E8_MOUSE	R	S	F	K	K	G	A	E
tr:D3YYN7:D3YYN7_MOUSE	R	S	F	K	K	G	A	E
sp:Q6PIC6:AT1A3_MOUSE	R	S	F	K	K	G	A	E
tr:Q91WH7:Q91WH7_MOUSE	R	S	F	K	K	G	A	E
sp:Q8VDN2:AT1A1_MOUSE	R	S	F	K	K	G	A	E
tr:Q3UHK5:Q3UHK5_MOUSE	R	S	F	K	K	G	A	E
sp:Q6PIE5:AT1A2_MOUSE	R	S	F	K	K	G	A	E
tr:E9QNX7:E9QNX7_MOUSE	R	S	F	K	K	G	A	E
sp:Q9Z1W8:AT12A_MOUSE	R	S	F	K	K	G	A	E
sp:Q9WV27:AT1A4_MOUSE	R	S	F	K	K	G	A	E
tr:Q8VCE0:Q8VCE0_MOUSE	R	S	F	K	K	G	A	E
tr:B5RIT8:B5RIT8_DROME	R	S	F	K	K	G	A	E
tr:E6PBW0:E6PBW0_DROME	R	S	F	K	K	G	A	E
tr:C8VUY7:C8VUY7_DROME	R	S	F	K	K	G	A	E
tr:E1JIR4:E1JIR4_DROME	R	S	F	K	K	G	A	E
sp:P13607:ATNA_DROME	R	S	F	K	K	G	A	E
tr:H9ZJM5:H9ZJM5_DROME	R	S	F	K	K	G	A	E
tr:P90735:P90735_CAEEL	R	S	F	K	K	G	A	E
tr:Q27461:Q27461_CAEEL	R	S	F	K	K	G	A	E
tr:E9P9X6:E9P9X6_YEASX	-	-	-	-	-	-	-	-
tr:E9P9X5:E9P9X5_YEASX	-	-	-	-	-	-	-	-
sp:Q01896:ATN2_YEAST	K	S	F	K	K	G	A	L
sp:P13587:ATN1_YEAST	K	S	F	K	K	G	A	L
sp:Q12691:ATN5_YEAST	K	S	F	K	K	G	A	L
tr:E9P9I4:E9P9I4_YEASX	K	S	F	K	K	G	A	L

protein	whole protein ident	whole protein simil	domain-based ident	domain-based simil	contact-based ident	contact-based simil
PDB:1mo8:chainA:P06685	1.0	1.0	1.0	1.0	1.0	1.0
tr:M0R116:M0R116_HUMAN	0.84	0.93	0.75	0.93	1.0	1.0
tr:B1AKY9:B1AKY9_HUMAN	0.85	0.95	0.71	0.91	1.0	1.0
sp:P13637:AT1A3_HUMAN	0.85	0.95	0.75	0.93	1.0	1.0
sp:P05023:AT1A1_HUMAN	0.97	0.99	0.95	0.99	1.0	1.0
sp:P50993:AT1A2_HUMAN	0.86	0.96	0.71	0.91	1.0	1.0
sp:Q13733:AT1A4_HUMAN	0.77	0.92	0.67	0.88	1.0	1.0
sp:P54707:AT12A_HUMAN	0.63	0.87	0.59	0.87	1.0	1.0
sp:P20648:ATP4A_HUMAN	0.61	0.87	0.58	0.87	1.0	1.0
sp:P06687:AT1A3_RAT	0.85	0.95	0.74	0.93	1.0	1.0
tr:Q5PQV3:Q5PQV3_RAT	0.76	0.92	0.65	0.87	1.0	1.0
sp:Q64541:AT1A4_RAT	0.77	0.92	0.65	0.87	1.0	1.0
sp:P06686:AT1A2_RAT	0.86	0.96	0.71	0.91	1.0	1.0
sp:P06685:AT1A1_RAT	1.0	1.0	1.0	1.0	1.0	1.0
tr:F1LRK1:F1LRK1_RAT	0.62	0.88	0.58	0.87	1.0	1.0
tr:G3V8S4:G3V8S4_RAT	0.63	0.88	0.6	0.87	1.0	1.0
tr:D3YVX6:D3YVX6_MOUSE	0.49	0.59	0.71	0.91	1.0	1.0
tr:Q8R0E8:Q8R0E8_MOUSE	0.64	0.71	0.74	0.93	1.0	1.0
tr:D3YYN7:D3YYN7_MOUSE	0.8	0.89	0.71	0.91	1.0	1.0
sp:Q6PIC6:AT1A3_MOUSE	0.85	0.95	0.74	0.93	1.0	1.0
tr:Q91WH7:Q91WH7_MOUSE	0.61	0.87	0.57	0.86	1.0	1.0
sp:Q8VDN2:AT1A1_MOUSE	1.0	1.0	0.99	1.0	1.0	1.0
tr:Q3UHK5:Q3UHK5_MOUSE	0.86	0.96	0.71	0.91	1.0	1.0
sp:Q6PIE5:AT1A2_MOUSE	0.86	0.96	0.71	0.91	1.0	1.0
tr:E9QNX7:E9QNX7_MOUSE	0.62	0.87	0.57	0.86	1.0	1.0
sp:Q9Z1W8:AT12A_MOUSE	0.63	0.88	0.59	0.88	1.0	1.0
sp:Q9WV27:AT1A4_MOUSE	0.78	0.92	0.65	0.87	1.0	1.0
tr:Q8VCE0:Q8VCE0_MOUSE	0.81	0.91	0.74	0.93	1.0	1.0
tr:B5RIT8:B5RIT8_DROME	0.6	0.76	0.63	0.9	1.0	1.0
tr:E6PBW0:E6PBW0_DROME	0.66	0.83	0.63	0.9	1.0	1.0
tr:C8VUY7:C8VUY7_DROME	0.74	0.91	0.63	0.9	1.0	1.0
tr:E1JIR4:E1JIR4_DROME	0.74	0.91	0.63	0.9	1.0	1.0
sp:P13607:ATNA_DROME	0.73	0.91	0.63	0.9	1.0	1.0
tr:H9ZJM5:H9ZJM5_DROME	0.73	0.91	0.63	0.9	1.0	1.0
tr:P90735:P90735_CAEEL	0.71	0.89	0.59	0.84	1.0	1.0
tr:Q27461:Q27461_CAEEL	0.71	0.89	0.59	0.84	1.0	1.0
tr:E9P9X6:E9P9X6_YEASX	0.02	0.07	0.0	0.0	0.0	0.0
tr:E9P9X5:E9P9X5_YEASX	0.02	0.07	0.0	0.0	0.0	0.0
sp:Q01896:ATN2_YEAST	0.25	0.6	0.23	0.62	0.75	0.81
sp:P13587:ATN1_YEAST	0.25	0.59	0.23	0.62	0.75	0.81
sp:Q12691:ATN5_YEAST	0.25	0.59	0.23	0.62	0.75	0.81
tr:E9P9I4:E9P9I4_YEASX	0.25	0.6	0.23	0.62	0.75	0.81

Atpalpha (FBgn0002921) associated phenotypes

auditory perception defective, bang sensitive, chemical resistant, chemical sensitive, circadian behavior defective, cold sensitive, conditional, dominant, fully, heat sensitive, hypoactive, lethal - all die before end of pupal stage, locomotor behavior defective, long lived, neuroanatomy defective, neurophysiology defective, paralytic, partially, partially lethal - majority die, partially lethal - majority live, phototaxis defective, rescuable maternal effect, short lived, some die during pupal stage

(Information from FlyBase)

Atpalpha (UniProt:P13607) annotation

Function: This is the catalytic component of the active enzyme, which catalyzes the hydrolysis of ATP coupled with the exchange of sodium and potassium ions across the plasma membrane. This action creates the electrochemical gradient of sodium and potassium ions, providing the energy for active transport of various nutrients. (PubMed:12598616, PubMed:2540956, PubMed:9648860).

Subunit: Composed of three subunits: alpha (catalytic), beta and gamma.

Subcellular location: Membrane; Multi-pass membrane protein.

Tissue specificity: High levels are found in some adult tissues: Malpighian tubules, indirect flight muscles, tubular leg muscles and throughout the nervous system (brain, optic lobes, retina and

ventral thoracic neuromere). Lower levels are detected at the posterior end where the reproductive organs and rectum are located. (PubMed:2540956, PubMed:9648860).
Rna editing: Modified$_{positions} = 429(PubMed : 12537569, PubMed : 17018572); Note = Partiallyedited.TargetofA$
Miscellaneous : $Ouabain - sensitiveelectrogenicionpump.$

$(InformationfromUniProt)$

eat-6 (WBGene00001137) associated phenotypes
DMPP resistant, acetylcholine synaptic transmission variant, aldicarb hypersensitive, dumpy, enzyme activity reduced, inhibitors of sodium potassium ATPase hypersensitive, levamisole hypersensitive, nicotine hypersensitive, nicotine resistant, pharyngeal contraction defective, pharyngeal physiology variant, pharyngeal pumping reduced, pharyngeal relaxation defective, postsynaptic region morphology variant, protein expression ectopic, protein modification variant, protein subcellular localization variant, reduced brood size, starved

(Information from WormBase)

Heat shock protein HSP 90-alpha

Best gene implication in ageing for this target family came from gene Q18688 annotated in UniProt release 2014_02. Annotation GO 8340 (determination of adult lifespan) was Inferred from Genetic Interaction

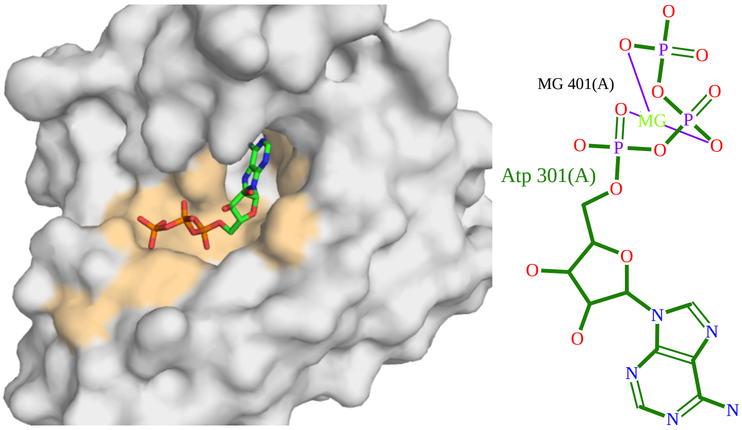

protein	amino acids contacts (binding site)
PDB:3t0z:chainA:P07900	E N A D M N L I G F G V G F T
tr:K9JA46:K9JA46_HUMAN	E N A D M N L I G F G V G F T
sp:P07900:HS90A_HUMAN	E N A D M N L I G F G V G F T
tr:Q6B437:Q6B437_RAT	E N A D M N L I G F G V G F T
sp:P82995:HS90A_RAT	E N A D M N L I G F G V G F T
tr:B7ZC50:B7ZC50_MOUSE	E N A D M N L I G F G V G F T
tr:Q8C2A9:Q8C2A9_MOUSE	E N A D M N L I G F G V G F T
tr:Q3TKB9:Q3TKB9_MOUSE	E N A D M N L I G F G V G F T
tr:Q3TKG0:Q3TKG0_MOUSE	E N A D M N L I G F G V G F T
sp:P07901:HS90A_MOUSE	E N A D M N L I G F G V G F T
tr:Q80Y52:Q80Y52_MOUSE	E N A D M N L I G F G V G F T
sp:P02828:HSP83_DROME	E N A D M N L I G F G V G F T
sp:Q18688:HSP90_CAEEL	E N A D M N L I G F G V G F T
sp:P02829:HSP82_YEAST	E N A D M N L I G F G V G F T

protein	whole protein ident	simil	domain-based ident	simil	contact-based ident	simil
PDB:3t0z:chainA:P07900	1.0	1.0	1.0	1.0	1.0	1.0
tr:K9JA46:K9JA46_HUMAN	1.0	1.0	1.0	1.0	1.0	1.0
sp:P07900:HS90A_HUMAN	1.0	1.0	1.0	1.0	1.0	1.0
tr:Q6B437:Q6B437_RAT	0.22	0.22	0.85	0.85	1.0	1.0
sp:P82995:HS90A_RAT	0.99	1.0	1.0	1.0	1.0	1.0
tr:B7ZC50:B7ZC50_MOUSE	0.27	0.27	0.86	0.86	1.0	1.0
tr:Q8C2A9:Q8C2A9_MOUSE	0.37	0.37	0.99	1.0	1.0	1.0
tr:Q3TKB9:Q3TKB9_MOUSE	0.75	0.76	0.99	1.0	1.0	1.0
tr:Q3TKG0:Q3TKG0_MOUSE	0.75	0.76	0.99	1.0	1.0	1.0
sp:P07901:HS90A_MOUSE	0.99	1.0	0.99	1.0	1.0	1.0
tr:Q80Y52:Q80Y52_MOUSE	0.99	1.0	0.99	1.0	1.0	1.0
sp:P02828:HSP83_DROME	0.77	0.92	0.85	0.96	1.0	1.0
sp:Q18688:HSP90_CAEEL	0.74	0.89	0.76	0.94	1.0	1.0
sp:P02829:HSP82_YEAST	0.6	0.84	0.68	0.9	1.0	1.0

Hsp83 (FBgn0001233) associated phenotypes
cell cycle defective, cell polarity defective, developmental rate defective, female semi-fertile, lethal - all die before end of pupal stage, lethal - all die during third instar larval stage, partially lethal, partially lethal - majority die, short lived

(Information from FlyBase)

Hsp83 (UniProt:P02828) annotation
Function: Molecular chaperone that promotes the maturation, structural maintenance and proper regulation of specific target proteins involved for instance in cell cycle control and signal transduction. Undergoes a functional cycle that is linked to its ATPase activity. This cycle probably induces conformational changes in the client proteins, thereby causing their activation. Interacts dynamically with various co-chaperones that modulate its substrate recognition, ATPase cycle and chaperone function. Together with Hop and piwi, mediates canalization, also known as developmental robustness, likely via epigenetic silencing of existing genetic variants and suppression of transposon-induced new genetic variation. Required for piRNA biogenesis by facilitating loading of piRNAs into PIWI proteins. (PubMed:21186352, PubMed:22902557).
Subunit: Homodimer (By similarity). Forms a complex with Hop and piwi; probably Hop mediates the interaction between piwi and Hsp83. Interacts with shu. (, PubMed:21186352, PubMed:22902557).
Subcellular location: Cytoplasm.
Induction: In contrast to other major heat shock proteins, this one is also expressed at normal growth temperatures. It is also developmentally expressed during oogenesis.
Domain: The TPR repeat-binding motif mediates interaction with TPR repeat-containing proteins.

(Information from UniProt)

daf-21 (WBGene00000915) associated phenotypes
chemotaxis variant, dauer constitutive, dauer recovery variant, larval lethal, lethal, odorant chemosensory response variant, sluggish, sterile

(Information from WormBase)

daf-21 (UniProt:Q18688) annotation
Function: Molecular chaperone that promotes the maturation, structural maintenance and proper regulation of specific target proteins involved for instance in cell cycle control and signal transduction. Undergoes a functional cycle that is linked to its ATPase activity. This cycle probably induces conformational changes in the client proteins, thereby causing their activation. Interacts dynamically with various co-chaperones that modulate its substrate recognition, ATPase cycle and chaperone function. By stabilizing the receptor-type guanylate cyclase daf-11 or another signal transduction component that regulates cGMP levels, plays a role in dauer formation and chemotaxis to non-volatile and volatile attractants detected by AWC sensory neurons

(PubMed:10790386, PubMed:7828815). Participates in the control of cell cycle progression at the prophase/metaphase transition in oocyte development by ensuring the activity of wee-1.3 kinase, which negatively regulates cdk-1 through its phosphorylation (PubMed:16466390). Regulates yap-1 nuclear export after heat shock treatment (PubMed:23396260). (PubMed:10790386, PubMed:16466390, PubMed:23396260, PubMed:7828815).
Subunit: Homodimer. (UniProtKB:P08238).
Subcellular location: Cytoplasm, perinuclear region (PubMed:12950278). Note=Perinuclear region of somatic cells.
Tissue specificity: In the embryo comma stage, expression is strongly detected in cells of the head region and less so in other areas. In early larvae, expressed in postembryonic germ cells derived from Z2 and Z3 cells and the head region, in both hermaphrodites and males. Under heat stress conditions, larval expression is not only detected in germ cells, but also all over the body. In adult hermaphrodites, expression is localized uniquely in the germ cells. (PubMed:12950278).
Developmental stage: Highly expressed throughout development. (PubMed:10790386).
Domain: The TPR repeat-binding motif mediates interaction with TPR repeat-containing proteins.

(Information from UniProt)

Hepatocyte growth factor receptor

Best gene implication in ageing for this target family came from gene Q2IBC7 via mapping the annotation from RGD 3082 annotated in RGD 2014-03-11. Annotation GO subterm of 7568 (aging) was Inferred from Expression Pattern

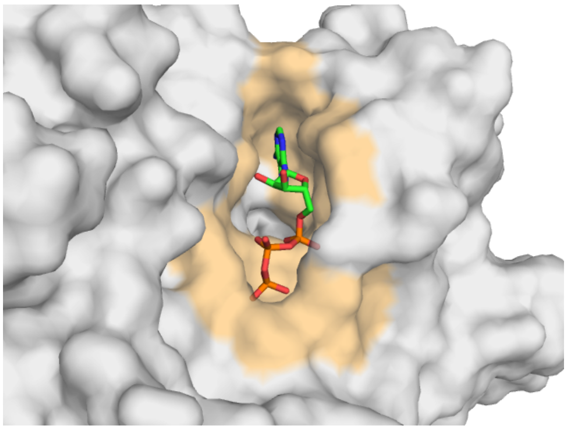

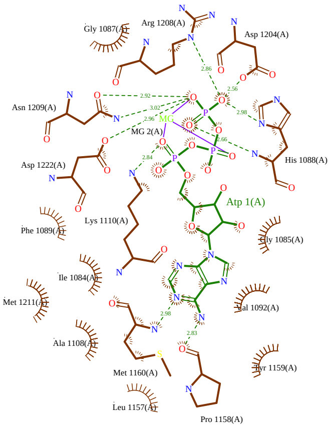

protein	amino acids contacts (binding site)
PDB:3dkc:chainA:P08581	I G G H F G V A K L P Y M D R N M D
tr:B4DLF5:B4DLF5_HUMAN	I G G H F G V A K L P Y M D R N M D
sp:P08581:MET_HUMAN	I G G H F G V A K L P Y M D R N M D
tr:Q2IBC7:Q2IBC7_RAT	I G G H F G V A K L P Y M D R N M D
tr:F8VQL0:F8VQL0_MOUSE	I G G H F G V A K L P Y M D R N M D
tr:Q6AHP3:Q6AHP3_CAEEL	I G G H Y G V V K T E Y M D R N L D
tr:H1AGA1:H1AGA1_CAEEL	I G G H Y G V V K T E Y M D R N L D
tr:H2KZU7:H2KZU7_CAEEL	I G G H Y G V V K T E Y M D R N L D

protein	whole protein ident	simil	domain-based ident	simil	contact-based ident	simil
PDB:3dkc:chainA:P08581	0.98	1.0	0.99	1.0	1.0	1.0
tr:B4DLF5:B4DLF5_HUMAN	0.69	0.69	1.0	1.0	1.0	1.0
sp:P08581:MET_HUMAN	1.0	1.0	1.0	1.0	1.0	1.0
tr:Q2IBC7:Q2IBC7_RAT	0.88	0.96	0.98	1.0	1.0	1.0
tr:F8VQL0:F8VQL0_MOUSE	0.89	0.96	0.99	1.0	1.0	1.0
tr:Q6AHP3:Q6AHP3_CAEEL	0.13	0.4	0.41	0.78	0.72	0.85
tr:H1AGA1:H1AGA1_CAEEL	0.13	0.4	0.41	0.78	0.72	0.85
tr:H2KZU7:H2KZU7_CAEEL	0.13	0.4	0.41	0.78	0.72	0.85

78 kDa glucose-regulated protein

Best gene implication in ageing for this target family came from gene P29844 via mapping the annotation from Ensembl FBgn0001218 via mapping the annotation from EntrezGene 32133 via mapping the annotation from GenAgeModels 0853 annotated in GenAge release 17.

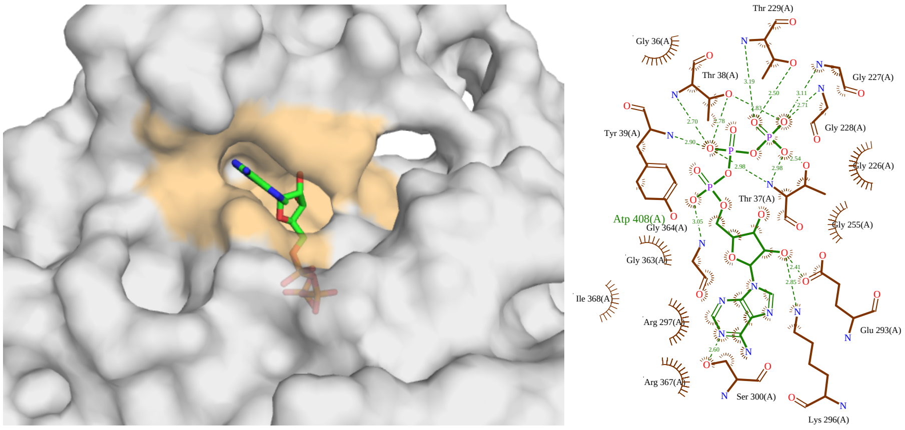

protein	amino acids contacts (binding site)																			
PDB:3ldl:chainA:P11021	G	T	T	Y	G	G	G	T	G	E	E	K	R	S	G	G	S	R	I	D
sp:P11021:GRP78_HUMAN	G	T	T	Y	G	G	G	T	G	E	E	K	R	S	G	G	S	R	I	D
sp:P06761:GRP78_RAT	G	T	T	Y	G	G	G	T	G	E	E	K	R	S	G	G	S	R	I	D
tr:Q3U7T8:Q3U7T8_MOUSE	G	T	T	Y	G	G	G	T	G	E	E	K	R	S	G	G	S	R	I	D
sp:P20029:GRP78_MOUSE	G	T	T	Y	G	G	G	T	G	E	E	K	R	S	G	G	S	R	I	D
sp:P29844:HSP7C_DROME	G	T	T	Y	G	G	G	T	G	E	E	K	R	S	G	G	S	R	I	D
tr:F3YDH0:F3YDH0_DROME	G	T	T	Y	G	G	G	T	G	E	E	K	R	S	G	G	S	R	I	D
sp:P20163:HSP7D_CAEEL	G	T	T	Y	G	G	G	T	G	E	E	K	R	S	G	G	S	R	I	D
sp:P27420:HSP7C_CAEEL	G	T	T	Y	G	G	G	T	G	E	E	K	R	S	G	G	S	R	I	D
sp:P10592:HSP72_YEAST	G	T	T	Y	G	G	G	T	G	E	E	K	R	S	G	G	S	R	I	D

protein	whole protein ident	simil	domain-based ident	simil	contact-based ident	simil
PDB:3ldl:chainA:P11021	1.0	1.0	1.0	1.0	1.0	1.0
sp:P11021:GRP78_HUMAN	1.0	1.0	1.0	1.0	1.0	1.0
sp:P06761:GRP78_RAT	0.98	1.0	1.0	1.0	1.0	1.0
tr:Q3U7T8:Q3U7T8_MOUSE	0.93	0.94	1.0	1.0	1.0	1.0
sp:P20029:GRP78_MOUSE	0.98	0.99	1.0	1.0	1.0	1.0
sp:P29844:HSP7C_DROME	0.8	0.94	0.9	0.98	1.0	1.0
tr:F3YDH0:F3YDH0_DROME	0.8	0.94	0.9	0.98	1.0	1.0
sp:P20163:HSP7D_CAEEL	0.75	0.92	0.86	0.96	1.0	1.0
sp:P27420:HSP7C_CAEEL	0.77	0.93	0.86	0.97	1.0	1.0
sp:P10592:HSP72_YEAST	0.6	0.83	0.71	0.91	1.0	1.0

Hsc70-3 (FBgn0001218) associated phenotypes

dominant, neuroanatomy defective, partially lethal - majority die

(Information from FlyBase)

Hsc70-3 (UniProt:P29844) annotation
Function: Probably plays a role in facilitating the assembly of multimeric protein complexes inside the ER.
Subcellular location: Endoplasmic reticulum lumen.

(Information from UniProt)

hsp-4 (WBGene00002008) associated phenotypes
energy expenditure variant, intestine dark, lipid hydrolysis regulation variant, organism starvation hypersensitive, thrashing reduced

(Information from WormBase)

hsp-4 (UniProt:P20163) annotation
Function: Probably plays a role in facilitating the assembly of multimeric protein complexes inside the ER.
Subcellular location: Endoplasmic reticulum lumen.

(Information from UniProt)

hsp-3 (UniProt:P27420) annotation
Function: Probably plays a role in facilitating the assembly of multimeric protein complexes inside the ER.
Subcellular location: Endoplasmic reticulum lumen.

(Information from UniProt)

Heat shock cognate 71 kDa protein

Best gene implication in ageing for this target family came from gene O97125 annotated in UniProt release 2014_02. Annotation GO 8340 (determination of adult lifespan) was Inferred from Mutant Phenotype

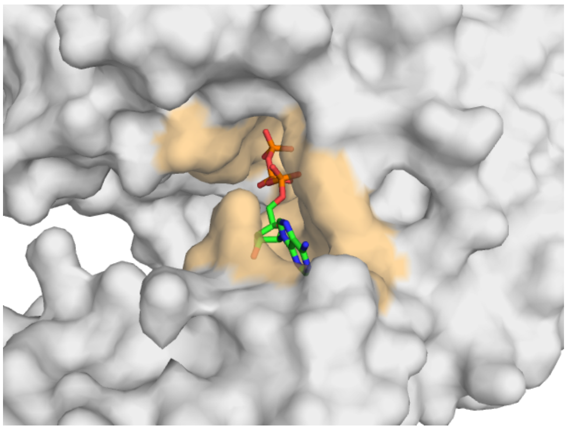

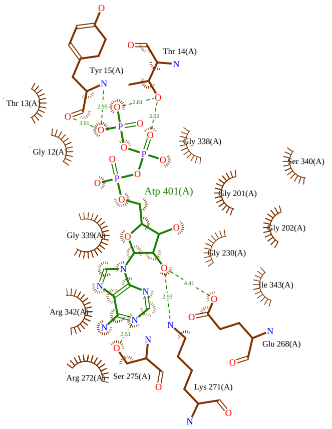

protein	amino acids contacts (binding site)
PDB:3fzf:chainA:P11142	G T T Y G G T G E K R S G G S R I D
tr:E7EP94:E7EP94_HUMAN	G T T Y G G T G E K R S G G S R I D
tr:E9PKE3:E9PKE3_HUMAN	G T T Y G G T G E K R S G G S R I D
sp:P08107:HSP71_HUMAN	G T T Y G G T G E K R S G G S R I D
sp:P11142:HSP7C_HUMAN	G T T Y G G T G E K R S G G S R I D
tr:A8K5I0:A8K5I0_HUMAN	G T T Y G G T G E K R S G G S R I D
tr:M0R8M9:M0R8M9_RAT	G T T Y G G T G E K R S G G S R I D
tr:F1LZI1:F1LZI1_RAT	G T T Y G G T G E K R S G G S R I D
tr:M0RCB1:M0RCB1_RAT	G T T Y G G T G E K R S G G S R I D
tr:Q3TB63:Q3TB63_MOUSE	G T T Y G G T G E K R S G G S R I D
tr:Q504P4:Q504P4_MOUSE	G T T Y G G T G E K R S G G S R I D
sp:P63017:HSP7C_MOUSE	G T T Y G G T G E K R S G G S R I D
sp:O97125:HSP68_DROME	G T T Y G G T G E K R S G G S R I D
sp:P29843:HSP7A_DROME	G T T Y G G T G E K R S G G S R I D
tr:F3YDI6:F3YDI6_DROME	G T T Y G G T G E K R S G G S R I D
sp:P82910:HSP70_DROME	G T T Y G G T G E K R S G G S R I D
sp:P02825:HSP71_DROME	G T T Y G G T G E K R S G G S R I D
tr:C7LA76:C7LA76_DROME	G T T Y G G T G E K R S G G S R I D
sp:P29844:HSP7C_DROME	G T T Y G G T G E K R S G G S R I D
sp:P11147:HSP7D_DROME	G T T Y G G T G E K R S G G S R I D
tr:C7LA75:C7LA75_DROME	G T T Y G G T G E K R S G G S R I D
tr:F3YDH0:F3YDH0_DROME	G T T Y G G T G E K R S G G S R I D
tr:F3YDH5:F3YDH5_DROME	G T T Y G G T G E K R S G G S R I D
tr:Q8IH88:Q8IH88_DROME	G T T Y G G T G E K R S G G S R I D
sp:P09446:HSP7A_CAEEL	G T T Y G G T G E K R S G G S R I D
sp:P10592:HSP72_YEAST	G T T Y G G T G E K R S G G S R I D

	whole protein		domain-based		contact-based	
protein	ident	simil	ident	simil	ident	simil
PDB:3fzf:chainA:P11142	1.0	1.0	1.0	1.0	1.0	1.0
tr:E7EP94:E7EP94_HUMAN	0.72	0.81	0.77	0.84	1.0	1.0
tr:E9PKE3:E9PKE3_HUMAN	0.97	0.97	0.94	0.94	1.0	1.0
sp:P08107:HSP71_HUMAN	0.84	0.94	0.9	0.97	1.0	1.0
sp:P11142:HSP7C_HUMAN	1.0	1.0	1.0	1.0	1.0	1.0
tr:A8K5I0:A8K5I0_HUMAN	0.84	0.94	0.9	0.97	1.0	1.0
tr:M0R8M9:M0R8M9_RAT	0.99	1.0	0.99	1.0	1.0	1.0
tr:F1LZI1:F1LZI1_RAT	1.0	1.0	0.99	1.0	1.0	1.0
tr:M0RCB1:M0RCB1_RAT	0.98	0.99	0.98	0.98	1.0	1.0
tr:Q3TB63:Q3TB63_MOUSE	0.71	0.71	1.0	1.0	1.0	1.0
tr:Q504P4:Q504P4_MOUSE	0.97	0.97	0.94	0.94	1.0	1.0
sp:P63017:HSP7C_MOUSE	1.0	1.0	1.0	1.0	1.0	1.0
sp:O97125:HSP68_DROME	0.7	0.89	0.84	0.95	1.0	1.0
sp:P29843:HSP7A_DROME	0.8	0.93	0.89	0.97	1.0	1.0
tr:F3YDI6:F3YDI6_DROME	0.73	0.9	0.84	0.95	1.0	1.0
sp:P82910:HSP70_DROME	0.73	0.9	0.84	0.95	1.0	1.0
sp:P02825:HSP71_DROME	0.73	0.9	0.84	0.95	1.0	1.0
tr:C7LA76:C7LA76_DROME	0.73	0.9	0.83	0.95	1.0	1.0
sp:P29844:HSP7C_DROME	0.59	0.83	0.71	0.92	1.0	1.0
sp:P11147:HSP7D_DROME	0.84	0.95	0.92	0.98	1.0	1.0
tr:C7LA75:C7LA75_DROME	0.84	0.95	0.92	0.98	1.0	1.0
tr:F3YDH0:F3YDH0_DROME	0.59	0.83	0.71	0.92	1.0	1.0
tr:F3YDH5:F3YDH5_DROME	0.77	0.9	0.89	0.97	1.0	1.0
tr:Q8IH88:Q8IH88_DROME	0.75	0.88	0.89	0.97	1.0	1.0
sp:P09446:HSP7A_CAEEL	0.84	0.94	0.9	0.97	1.0	1.0
sp:P10592:HSP72_YEAST	0.75	0.92	0.84	0.95	1.0	1.0

Hsp68 (FBgn0001230) associated phenotypes

chemical resistant, long lived, neuroanatomy defective, partially lethal - majority die, some die during pupal stage, starvation stress response defective

(Information from FlyBase)

Hsc70-1 (FBgn0001216) associated phenotypes
partially lethal - majority die, some die during pupal stage

(Information from FlyBase)

Hsc70-1 (UniProt:P29843) annotation
Developmental stage: Heat shock cognate proteins are expressed constitutively during normal development.

(Information from UniProt)

Hsp70Aa (UniProt:P82910) annotation
Induction: Heat shock induces the synthesis of seven proteins at five otherwise inactive sites in the polytene chromosomes of fruit fly larvae. Two separate sites, producing two and three copies, respectively, code for the 70 kDa protein.
Miscellaneous: There are two copies of the gene coding for this protein at chromosome locus 87A7.

(Information from UniProt)

Hsp70Ab (UniProt:P02825) annotation
Induction: Heat shock induces the synthesis of seven proteins at five otherwise inactive sites in the polytene chromosomes of fruit fly larvae. Two separate sites, producing two and three copies, respectively, code for the 70 kDa protein.
Miscellaneous: There are two copies of the gene coding for this protein at chromosome locus 87A7.

(Information from UniProt)

Hsc70-3 (FBgn0001218) associated phenotypes
dominant, neuroanatomy defective, partially lethal - majority die

(Information from FlyBase)

Hsc70-3 (UniProt:P29844) annotation
Function: Probably plays a role in facilitating the assembly of multimeric protein complexes inside the ER.
Subcellular location: Endoplasmic reticulum lumen.

(Information from UniProt)

Hsc70-4 (UniProt:P11147) annotation
Subcellular location: Cytoplasm, perinuclear region. Nucleus. Note=Localized to a meshwork of cytoplasmic fibers around the nucleus. Translocates to the nucleus after thermal stress.
Developmental stage: Heat shock cognate proteins are expressed constitutively during normal development.

(Information from UniProt)

hsp-1 (WBGene00002005) associated phenotypes
lethal, sterile

(Information from WormBase)

NAD-dependent malic enzyme, mitochondrial

Best gene implication in ageing for this target family came from gene Q9NIW0 annotated in UniProt release 2014_02. Annotation GO 8340 (determination of adult lifespan) was Inferred from Mutant Phenotype

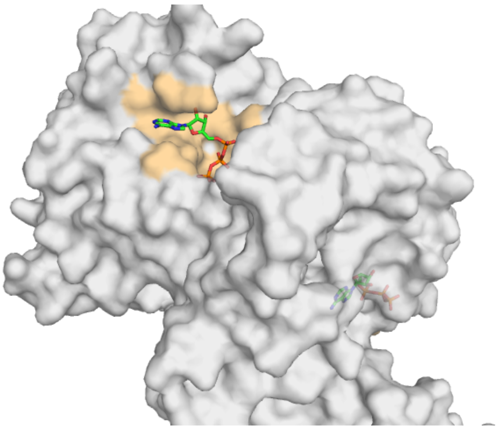

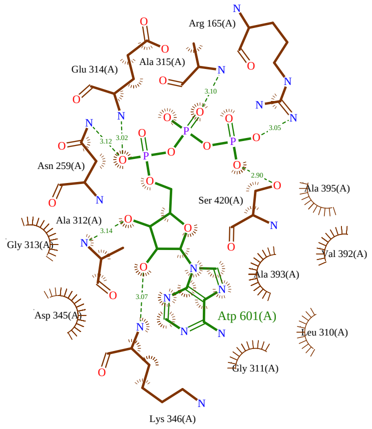

protein	amino acids contacts (binding site)
PDB:1gz3:chainA:P23368	H K R L G I R Q D N L G A G E A F D K V A G A L S I L N V Y Q
PDB:1gz4:chainA:P23368	H K R L G I R R D N L G A G E A F D K V A G A L S I L N R Y R
PDB:1pj4:chainA:P23368	H K R L G I R R D N L G A G E A F D K V A G A L S I L N R Y R
sp:P23368:MAOM_HUMAN	H K R L G I R R D N L G A G E A F D K V A G A L S I L N R Y R
tr:D3ZJH9:D3ZJH9_RAT	H K R L G I Q K D N L G A G E A F D K V A G A L S I L E R Y R
sp:Q99KE1:MAOM_MOUSE	H K R L G I Q K D N L G A G E A F D K V A G A L S I L E R Y R
tr:H7BWR6:H7BWR6_DROME	D R R L G I K Q R N L G A G E A V D S A A A Q A S L C G V F Q
tr:Q9NIW0:Q9NIW0_DROME	D R R L G I K Q R N L G A G E A V D S A A A Q A S L C G V F Q
tr:H7BWR4:H7BWR4_DROME	D R R L G I K Q R N L G A G E A V D S A A A Q A S L C G V F Q
tr:H7BWU6:H7BWU6_DROME	D R R L G I K Q R N L G A G E A V D S A A A Q A S L C G V F Q
tr:H7BWS3:H7BWS3_DROME	D R R L G I K Q R N L G A G E A V D S A A A Q A S L C G V F Q
tr:H7BWS2:H7BWS2_DROME	D R R L G I K Q R N L G A G E A V D S A A A Q A S L C G V F Q
tr:Q2QBM1:Q2QBM1_DROME	D R R L G I K Q R N L G A G E A V D S A A A Q A S L C G V F Q
tr:H7BWS7:H7BWS7_DROME	D R R L G I K Q R N L G A G E A V D S A A A Q A S L C G V F Q
tr:H7BWS6:H7BWS6_DROME	D R R L G I K Q R N L G A G E A V D S A A A Q A S L C G V F Q
tr:H7BWU8:H7BWU8_DROME	D R R L G I K Q R N L G A G E A V D S A A A Q A S L C G V F Q
tr:Q9U1J1:Q9U1J1_DROME	D R R L G I K Q R N L G A G E A V D S A A A Q A S L C G V F Q
tr:Q9NIW1:Q9NIW1_DROME	D R R L G I K Q R N L G A G E A V D S A A A Q A S L C G V F Q
tr:Q9VG32:Q9VG32_DROME	D R R L G I K Q R N L G A G E A V D S A A A Q A S L C G V F Q
tr:Q9NIW2:Q9NIW2_DROME	D R R L G I K Q R N L G A G E A V D S A A A Q A S L C G V F Q
tr:Q9VG31:Q9VG31_DROME	D R R L G I K Q R N L G A G E A V D S A A A Q A S L C G V F Q
tr:Q9U296:Q9U296_CAEEL	N R R L G I R W K N L G A G G A V D I A S T V A S V L R L F N
sp:P36013:MAOM_YEAST	D D G L G I H R K V Y G A G S A M D R C S T Q A S V L R I W Q

protein	whole protein ident	simil	domain-based ident	simil	contact-based ident	simil
PDB:1gz3:chainA:P23368	0.99	1.0	0.99	1.0	1.0	1.0
PDB:1gz4:chainA:P23368	1.0	1.0	1.0	1.0	0.9	0.94
PDB:1pj4:chainA:P23368	1.0	1.0	1.0	1.0	0.9	0.94
sp:P23368:MAOM_HUMAN	1.0	1.0	1.0	1.0	0.9	0.94
tr:D3ZJH9:D3ZJH9_RAT	0.89	0.97	0.9	0.97	0.84	0.91
sp:Q99KE1:MAOM_MOUSE	0.88	0.96	0.9	0.97	0.84	0.91
tr:H7BWR6:H7BWR6_DROME	0.47	0.77	0.5	0.82	0.55	0.69
tr:Q9NIW0:Q9NIW0_DROME	0.46	0.76	0.5	0.82	0.55	0.69
tr:H7BWR4:H7BWR4_DROME	0.46	0.76	0.5	0.82	0.55	0.69
tr:H7BWU6:H7BWU6_DROME	0.45	0.76	0.5	0.82	0.55	0.69
tr:H7BWS3:H7BWS3_DROME	0.46	0.76	0.5	0.82	0.55	0.69
tr:H7BWS2:H7BWS2_DROME	0.46	0.76	0.5	0.82	0.55	0.69
tr:Q2QBM1:Q2QBM1_DROME	0.46	0.76	0.5	0.82	0.55	0.69
tr:H7BWS7:H7BWS7_DROME	0.45	0.76	0.5	0.82	0.55	0.69
tr:H7BWS6:H7BWS6_DROME	0.45	0.76	0.5	0.82	0.55	0.69
tr:H7BWU8:H7BWU8_DROME	0.46	0.76	0.5	0.82	0.55	0.69
tr:Q9U1J1:Q9U1J1_DROME	0.44	0.74	0.48	0.8	0.55	0.69
tr:Q9NIW1:Q9NIW1_DROME	0.36	0.61	0.5	0.82	0.55	0.69
tr:Q9VG32:Q9VG32_DROME	0.36	0.61	0.5	0.82	0.55	0.69
tr:Q9NIW2:Q9NIW2_DROME	0.36	0.61	0.5	0.82	0.55	0.69
tr:Q9VG31:Q9VG31_DROME	0.36	0.61	0.5	0.82	0.55	0.69
tr:Q9U296:Q9U296_CAEEL	0.47	0.78	0.51	0.84	0.45	0.65
sp:P36013:MAOM_YEAST	0.3	0.65	0.34	0.74	0.35	0.58

UniProt:H7BWR6 annotation
Cofactor: Mg(2+) Evidence=(PIRSR:PIRSR000106-3); Name=Mn(2+); Xref=ChEBI:CHEBI:29035; Evidence=(PIRSR:PIRSR000106-3); Note=Divalent metal cations. Prefers magnesium or manganese. (PIRSR:PIRSR000106-3);

(Information from UniProt)

Men (FBgn0002719) associated phenotypes
fast, intermediate, long lived, poor, sleep defective

(Information from FlyBase)

Men (UniProt:Q9NIW0) annotation
Cofactor: Mg(2+) Evidence=(PIRSR:PIRSR000106-3); Name=Mn(2+); Xref=ChEBI:CHEBI:29035; Evidence=(PIRSR:PIRSR000106-3); Note=Divalent metal cations. Prefers magnesium or manganese. (PIRSR:PIRSR000106-3);

(Information from UniProt)

UniProt:H7BWR4 annotation
Cofactor: Mg(2+) Evidence=(PIRSR:PIRSR000106-3); Name=Mn(2+); Xref=ChEBI:CHEBI:29035; Evidence=(PIRSR:PIRSR000106-3); Note=Divalent metal cations. Prefers magnesium or manganese. (PIRSR:PIRSR000106-3);

(Information from UniProt)

UniProt:H7BWU6 annotation
Cofactor: Mg(2+)Name=Mn(2+); Xref=ChEBI:CHEBI:29035; Note=Divalent metal cations. Prefers magnesium or manganese.;

(Information from UniProt)

UniProt:H7BWS3 annotation
Cofactor: Mg(2+) Evidence=(PIRSR:PIRSR000106-3); Name=Mn(2+); Xref=ChEBI:CHEBI:29035; Evidence=(PIRSR:PIRSR000106-3); Note=Divalent metal cations. Prefers magnesium or manganese. (PIRSR:PIRSR000106-3);

(Information from UniProt)

UniProt:H7BWS2 annotation
Cofactor: Mg(2+) Evidence=(PIRSR:PIRSR000106-3); Name=Mn(2+); Xref=ChEBI:CHEBI:29035; Evidence=(PIRSR:PIRSR000106-3); Note=Divalent metal cations. Prefers magnesium or manganese. (PIRSR:PIRSR000106-3);

(Information from UniProt)

Men (UniProt:Q2QBM1) annotation
Cofactor: Mg(2+)Name=Mn(2+); Xref=ChEBI:CHEBI:29035; Note=Divalent metal cations. Prefers magnesium or manganese.;

(Information from UniProt)

UniProt:H7BWS7 annotation
Cofactor: Mg(2+) Evidence=(PIRSR:PIRSR000106-3); Name=Mn(2+); Xref=ChEBI:CHEBI:29035; Evidence=(PIRSR:PIRSR000106-3); Note=Divalent metal cations. Prefers magnesium or manganese. (PIRSR:PIRSR000106-3);

(Information from UniProt)

UniProt:H7BWS6 annotation
Cofactor: Mg(2+) Evidence=(PIRSR:PIRSR000106-3); Name=Mn(2+); Xref=ChEBI:CHEBI:29035; Evidence=(PIRSR:PIRSR000106-3); Note=Divalent metal cations. Prefers magnesium or manganese. (PIRSR:PIRSR000106-3);

(Information from UniProt)

UniProt:H7BWU8 annotation
Cofactor: Mg(2+) Evidence=(PIRSR:PIRSR000106-3); Name=Mn(2+); Xref=ChEBI:CHEBI:29035; Evidence=(PIRSR:PIRSR000106-3); Note=Divalent metal cations. Prefers magnesium or manganese. (PIRSR:PIRSR000106-3);

(Information from UniProt)

Men (UniProt:Q9U1J1) annotation
Cofactor: Mg(2+) Evidence=(PIRSR:PIRSR000106-3); Name=Mn(2+); Xref=ChEBI:CHEBI:29035; Evidence=(PIRSR:PIRSR000106-3); Note=Divalent metal cations. Prefers magnesium or manganese. (PIRSR:PIRSR000106-3);

(Information from UniProt)

men-1 (UniProt:Q9U296) annotation
Cofactor: Mg(2+) Evidence=(PIRSR:PIRSR000106-3); Name=Mn(2+); Xref=ChEBI:CHEBI:29035; Evidence=(PIRSR:PIRSR000106-3); Note=Divalent metal cations. Prefers magnesium or manganese. (PIRSR:PIRSR000106-3);

(Information from UniProt)

Insulin-degrading enzyme

Best gene implication in ageing for this target family came from gene P14735 annotated in UniProt release 2014_02. Annotation GO 8340 (determination of adult lifespan) was Inferred from Direct Assay

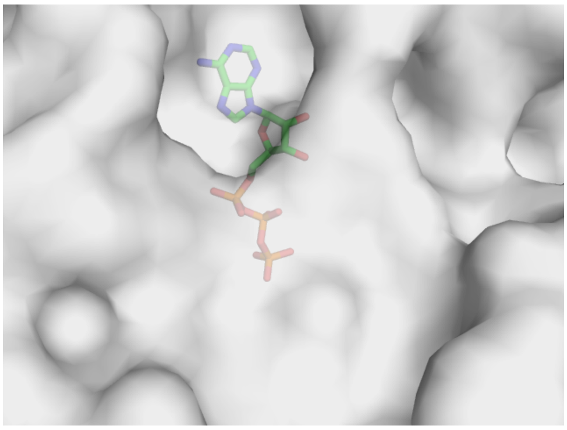

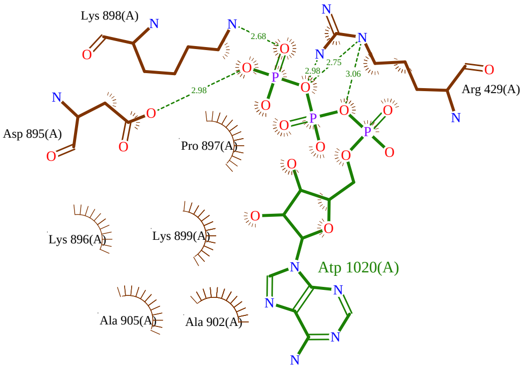

protein	amino acids contacts (binding site)								
PDB:3tuv:chainA:P35559	R	D	K	P	K	K	S	A	A
sp:P14735:IDE_HUMAN	R	D	K	P	K	K	S	A	A
sp:P35559:IDE_RAT	R	D	K	P	K	K	S	A	A
sp:P22817:IDE_DROME	Q	E	K	P	K	T	F	Q	S
tr:Q6A598:Q6A598_CAEEL	Q	E	K	P	K	T	S	S	R
tr:Q17592:Q17592_CAEEL	Q	E	K	P	K	T	S	N	K
tr:G5ED87:G5ED87_CAEEL	Q	E	K	P	K	T	S	C	K
tr:Q8IA89:Q8IA89_CAEEL	Q	E	K	P	K	T	S	S	R
tr:Q8MXK1:Q8MXK1_CAEEL	Q	E	K	P	K	T	S	S	R
tr:O16249:O16249_CAEEL	Q	E	K	P	K	T	S	S	R
sp:Q06010:STE23_YEAST	S	Q	K	F	K	N	A	E	A

protein	whole protein ident	whole protein simil	domain-based ident	domain-based simil	contact-based ident	contact-based simil
PDB:3tuv:chainA:P35559	1.0	1.0	1.0	1.0	1.0	1.0
sp:P14735:IDE_HUMAN	0.95	0.99	0.96	0.99	1.0	1.0
sp:P35559:IDE_RAT	1.0	1.0	1.0	1.0	1.0	1.0
sp:P22817:IDE_DROME	0.44	0.79	0.42	0.8	0.33	0.51
tr:Q6A598:Q6A598_CAEEL	0.37	0.71	0.35	0.69	0.44	0.61
tr:Q17592:Q17592_CAEEL	0.36	0.71	0.32	0.72	0.44	0.6
tr:G5ED87:G5ED87_CAEEL	0.37	0.75	0.35	0.77	0.44	0.51
tr:Q8IA89:Q8IA89_CAEEL	0.38	0.74	0.37	0.77	0.44	0.61
tr:Q8MXK1:Q8MXK1_CAEEL	0.38	0.76	0.37	0.77	0.44	0.61
tr:O16249:O16249_CAEEL	0.38	0.75	0.37	0.77	0.44	0.61
sp:Q06010:STE23_YEAST	0.36	0.76	0.34	0.76	0.33	0.5

Ide (FBgn0001247) associated phenotypes

decreased cell number, decreased cell size, developmental rate defective, large body, long lived, nutrition conditional, partially lethal - majority die, short lived, size defective, small body

(Information from FlyBase)

Ide (UniProt:P22817) annotation

Function: Can cleave insulin and TGF-alpha. (PubMed:3139025).
Cofactor: Zn(2+)Note=Binds 1 zinc ion per subunit. ;
Biophysicochemical properties: pH dependence: Optimum pH is 7-8. (PubMed:3139025); Temperature dependence: Optimum temperature is 37 degrees Celsius. (PubMed:3139025);
Induction: Inhibited by bacitracin and sulfhydryl-specific reagents. (PubMed:3139025).

(Information from UniProt)

Ubiquitin-like modifier-activating enzyme ATG7

Best gene implication in ageing for this target family came from gene A1ZBA9 annotated in UniProt release 2014_02. Annotation GO 8340 (determination of adult lifespan) was Inferred from Mutant Phenotype

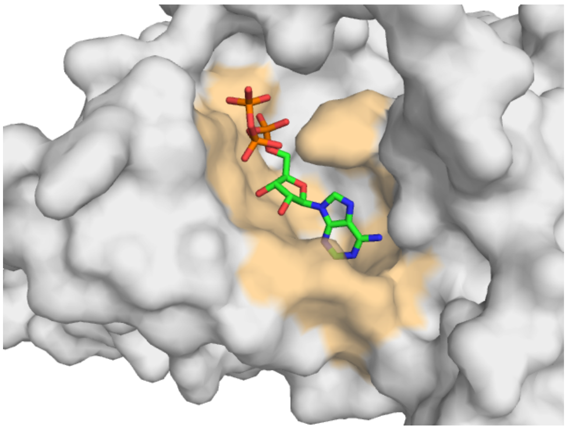

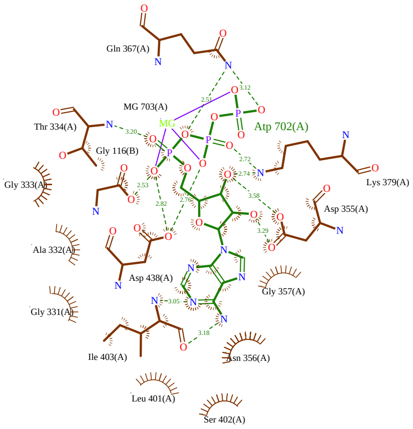

protein	amino acids contacts (binding site)													
PDB:3vh4:chainA:P38862	G	A	G	T	D	N	G	N	Q	K	L	S	I	D	S
sp:O95352:ATG7_HUMAN	G	A	G	T	D	N	A	N	Q	K	M	S	I	D	S
sp:Q641Y5:ATG7_RAT	G	A	G	T	D	N	A	N	Q	K	M	S	I	D	S
tr:S4R2D5:S4R2D5_MOUSE	G	A	G	T	D	N	A	N	Q	K	M	S	I	D	S
tr:S4R2T5:S4R2T5_MOUSE	G	A	G	T	D	N	A	N	Q	K	M	S	I	D	S
sp:Q9D906:ATG7_MOUSE	G	A	G	T	D	N	A	N	Q	K	M	S	I	D	S
tr:A1ZBA9:A1ZBA9_DROME	G	A	G	T	D	S	G	N	Q	K	L	E	I	D	S
tr:Q7JY94:Q7JY94_DROME	G	A	G	T	D	S	G	N	Q	K	L	E	I	D	S
tr:G5EBK4:G5EBK4_CAEEL	G	A	G	T	D	N	S	N	Q	K	L	T	V	D	A
sp:P38862:ATG7_YEAST	G	A	G	T	D	N	G	N	Q	K	L	S	I	D	S

	whole protein		domain-based		contact-based	
protein	ident	simil	ident	simil	ident	simil
PDB:3vh4:chainA:P38862	1.0	1.0	1.0	1.0	1.0	1.0
sp:O95352:ATG7_HUMAN	0.35	0.67	0.48	0.72	0.87	0.95
sp:Q641Y5:ATG7_RAT	0.35	0.67	0.48	0.72	0.87	0.95
tr:S4R2D5:S4R2D5_MOUSE	0.33	0.61	0.48	0.72	0.87	0.95
tr:S4R2T5:S4R2T5_MOUSE	0.34	0.64	0.44	0.66	0.87	0.95
sp:Q9D906:ATG7_MOUSE	0.36	0.67	0.48	0.72	0.87	0.95
tr:A1ZBA9:A1ZBA9_DROME	0.3	0.55	0.47	0.72	0.87	0.92
tr:Q7JY94:Q7JY94_DROME	0.35	0.68	0.47	0.72	0.87	0.92
tr:G5EBK4:G5EBK4_CAEEL	0.33	0.66	0.45	0.73	0.73	0.84
sp:P38862:ATG7_YEAST	1.0	1.0	1.0	1.0	1.0	1.0

Atg7 (FBgn0034366) associated phenotypes

RU486 conditional, chemical sensitive, decreased cell death, immune response defective, long lived, memory defective, neuroanatomy defective, nutrition conditional, progressive, short lived, somatic clone, stress response defective

(Information from FlyBase)

atg-7 (WBGene00010882) associated phenotypes

P granule localization defective, dead eggs laid, larval lethal, lethal, sterile

(Information from WormBase)

DNA mismatch repair protein Mlh1

Best gene implication in ageing for this target family came from gene P40692 via mapping the annotation from Ensembl ENSG00000076242 via mapping the annotation from EntrezGene 4292 via mapping the annotation from GenAgeHuman 0243 annotated in GenAge release 17.

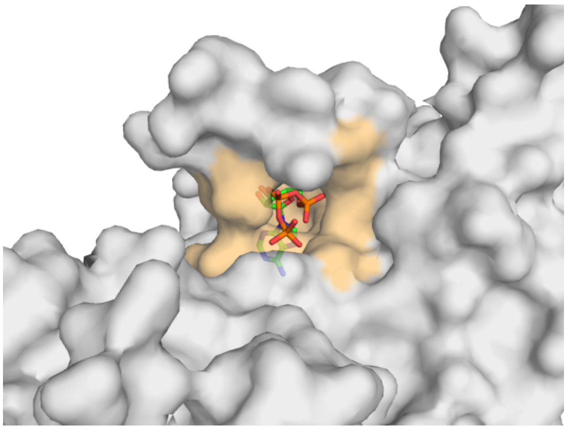

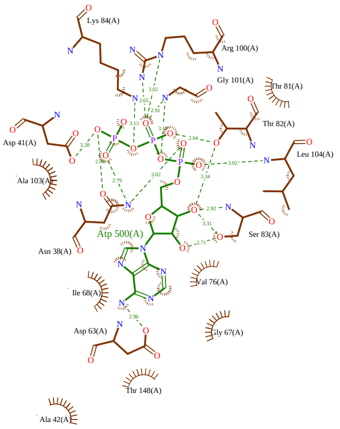

protein	amino acids contacts (binding site)																
PDB:3na3:chainA:P40692	E	N	D	A	D	G	I	V	T	T	S	K	R	G	E	A	L	T
sp:P40692:MLH1_HUMAN	E	N	D	A	D	G	I	V	T	T	S	K	R	G	E	A	L	T
tr:Q5GJ64:Q5GJ64_HUMAN	E	N	D	A	D	G	I	V	T	T	S	K	R	G	E	A	L	T
tr:F1LSD8:F1LSD8_RAT	E	N	D	A	D	G	I	V	T	T	S	K	R	G	E	A	L	T
sp:Q9JK91:MLH1_MOUSE	E	N	D	A	D	G	I	V	T	T	S	K	R	G	E	A	L	T
tr:A1Z7C1:A1Z7C1_DROME	E	N	D	A	D	G	I	V	T	T	S	K	R	G	E	A	L	T
tr:O61917:O61917_DROME	E	N	D	A	D	G	I	V	T	T	S	K	R	G	E	A	L	T
tr:Q9XU10:Q9XU10_CAEEL	E	N	D	A	D	G	I	V	A	T	S	K	R	G	E	A	L	T
sp:P38920:MLH1_YEAST	E	N	D	A	D	G	I	L	T	T	S	K	R	G	E	A	L	T

protein	whole protein ident	whole protein simil	domain-based ident	domain-based simil	contact-based ident	contact-based simil
PDB:3na3:chainA:P40692	1.0	1.0	1.0	1.0	1.0	1.0
sp:P40692:MLH1_HUMAN	1.0	1.0	1.0	1.0	1.0	1.0
tr:Q5GJ64:Q5GJ64_HUMAN	1.0	1.0	1.0	1.0	1.0	1.0
tr:F1LSD8:F1LSD8_RAT	0.88	0.96	0.96	0.99	1.0	1.0
sp:Q9JK91:MLH1_MOUSE	0.88	0.96	0.95	0.99	1.0	1.0
tr:A1Z7C1:A1Z7C1_DROME	0.45	0.73	0.68	0.91	1.0	1.0
tr:O61917:O61917_DROME	0.44	0.72	0.68	0.91	1.0	1.0
tr:Q9XU10:Q9XU10_CAEEL	0.3	0.67	0.54	0.85	0.94	0.97
sp:P38920:MLH1_YEAST	0.36	0.72	0.6	0.87	0.94	0.98

Cyclin-dependent kinase 7

Best gene implication in ageing for this target family came from gene Q3THG5 via mapping the annotation from Ensembl ENSMUSG00000069089 via mapping the annotation from EntrezGene 12572 via mapping the annotation from GenAgeModels 1813 annotated in GenAge release 17.

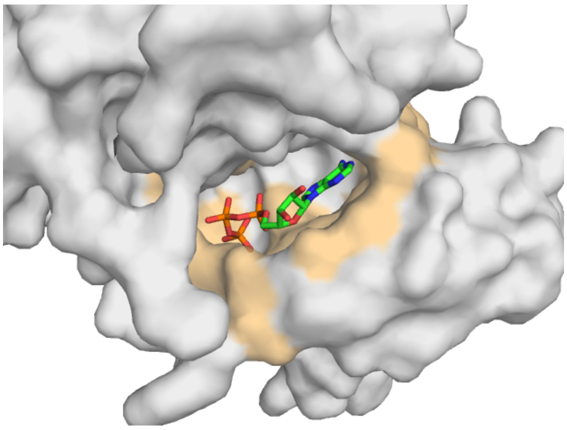

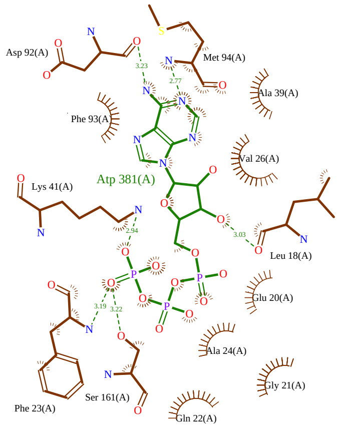

protein	amino acids contacts (binding site)														
PDB:1ua2:chainA:P50613	L	E	G	Q	F	A	V	A	K	D	F	M	K	N	S
tr:D6RAD4:D6RAD4_HUMAN	L	E	G	Q	F	A	V	A	K	D	F	M	K	N	S
sp:P50613:CDK7_HUMAN	L	E	G	Q	F	A	V	A	K	D	F	M	K	N	S
tr:M0RD95:M0RD95_RAT	L	E	G	Q	F	A	V	A	K	N	H	L	K	N	S
tr:F1LQC8:F1LQC8_RAT	L	E	G	Q	F	A	V	A	K	N	H	L	K	N	S
tr:Q3THG5:Q3THG5_MOUSE	L	E	G	Q	F	A	V	A	K	D	F	M	K	N	S
sp:Q03147:CDK7_MOUSE	L	E	G	Q	F	A	V	A	K	D	F	M	K	N	S
tr:Q24216:Q24216_DROME	L	E	G	Q	F	A	V	A	K	D	F	M	K	N	S
tr:G5EFV5:G5EFV5_CAEEL	L	E	G	Q	F	A	V	A	K	D	F	M	K	N	F
sp:P06242:KIN28_YEAST	V	E	G	T	Y	A	V	A	K	E	F	L	K	N	A

protein	whole protein ident	whole protein simil	domain-based ident	domain-based simil	contact-based ident	contact-based simil
PDB:1ua2:chainA:P50613	1.0	1.0	1.0	1.0	1.0	1.0
tr:D6RAD4:D6RAD4_HUMAN	0.89	0.89	0.87	0.87	1.0	1.0
sp:P50613:CDK7_HUMAN	1.0	1.0	1.0	1.0	1.0	1.0
tr:M0RD95:M0RD95_RAT	0.65	0.7	0.74	0.8	0.8	0.91
tr:F1LQC8:F1LQC8_RAT	0.91	0.98	0.91	0.98	0.8	0.91
tr:Q3THG5:Q3THG5_MOUSE	0.95	0.98	0.97	0.99	1.0	1.0
sp:Q03147:CDK7_MOUSE	0.95	0.98	0.97	0.99	1.0	1.0
tr:Q24216:Q24216_DROME	0.65	0.87	0.72	0.91	1.0	1.0
tr:G5EFV5:G5EFV5_CAEEL	0.53	0.78	0.61	0.86	0.93	0.9
sp:P06242:KIN28_YEAST	0.41	0.73	0.47	0.82	0.6	0.83

Cdk7 (FBgn0263237) associated phenotypes

decreased cell death, germline clone, heat sensitive, mitotic cell cycle defective, partially lethal - majority die, somatic clone, some die during pupal stage, temperature conditional

(Information from FlyBase)

cdk-7 (UniProt:G5EFV5) annotation

Function: Serine/threonine kinase involved in cell cycle control and in RNA polymerase II-mediated RNA transcription. Required for maintaining chromosome ploidy. May phosphorylate the large subunit of RNA polymerase II, ama-1. (PubMed:11960010).

Subunit: Catalytic component which, in association with cyclin H (cyh-1) and mat1, is likely to form the CAK complex. (UniProtKB:P50613).

Disruption phenotype: RNAi-mediated knockdown results in an arrest at approximately 50-cell embryonic stage. The phenotype is more severe when done in a cdk7(ax224) mutant background where the embryonic arrest occurs at the 1-cell stage due to an arrest in early meiosis. (PubMed:11960010).

(Information from UniProt)

5'-AMP-activated protein kinase subunit gamma-1

Best gene implication in ageing for this target family came from gene P12904 annotated in UniProt release 2014_02. Annotation GO subterm of 7568 (aging) was Inferred from Mutant Phenotype

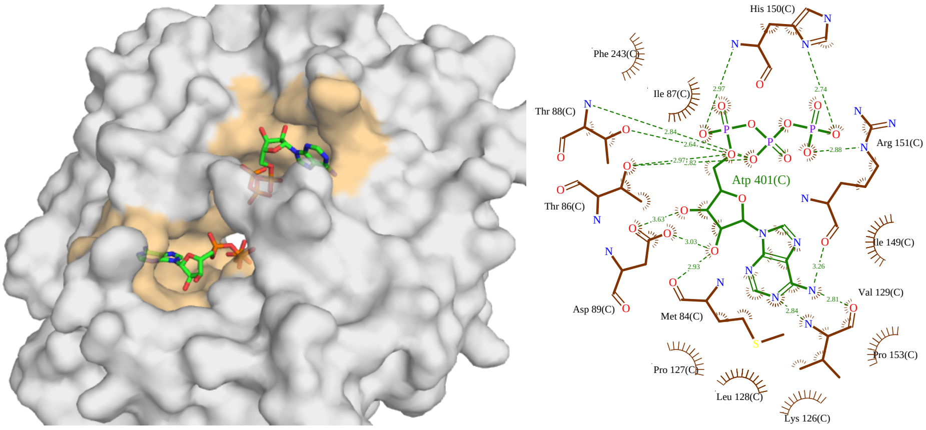

protein	amino acids contacts (binding site)
PDB:2v92:chainE:P80385	R M T I T D Q K P L V I H R P H K T N I A V S A L P I S K F D R F G V L V H R
PDB:2v9j:chainE:P80385	R M T I T D Q K P L V I H R P H K T N I A V S A L P I S K F D R F G V L V H R
PDB:4eag:chainC:P80385	R M T I T D Q K P L V I H R P H K T N I A V S A L P I S K F D R - G V L V H R
PDB:4eaj:chainC:P80385	R M T I T D - K P L V I H R P H K T N I A V S A L P I S K F D R F G V L V H R
PDB:4eak:chainC:P80385	R M T I T D Q K P L V I H R P H K T N I A V S A L P I S K F D R - - V L V H R
tr:F8VYY9:F8VYY9_HUMAN	R M T I T D Q K P L V I H R P H K T N I A V S A L P I S K F D R F G V L V H R
sp:P54619:AAKG1_HUMAN	R M T I T D Q K P L V I H R P H K T N I A V S A L P I S K F D R F G V L V H R
sp:P80385:AAKG1_RAT	R M T I T D Q K P L V I H R P H K T N I A V S A L P I S K F D R F G V L V H R
sp:O54950:AAKG1_MOUSE	R M T I T D Q K P L V I H R P H K T N I A V S A L P I S K F D R F G V L V H R
tr:Q3TWR3:Q3TWR3_MOUSE	R M T I T D Q K P L V I H R P H K T N I A V S A L P I S K F D R F G V L V H R
tr:Q7KNS9:Q7KNS9_DROME	R M T I T D H M P L V I H R P H K T N I E V S A L P I A K F D R F G V Q V H R
tr:Q8IGX0:Q8IGX0_DROME	R M T I T D H M P L V I H R P H K T N I E V S A L P I A K F D R F G V Q V H R
tr:Q0KI34:Q0KI34_DROME	R M T I T D H M P L V I H R P H K T N I E V S A L P I A K F D R F G V Q V H R
tr:A1A703:A1A703_DROME	R M T I T D H M P L V I H R P H K T N I E V S A L P I A K F D R F G V Q V H R
tr:A1A6Y3:A1A6Y3_DROME	R M T I T D H M P L V I H R P H K T N I E V S A L P I A K F D R F G V Q V H R
tr:O96613:O96613_DROME	R M T I T D H M P L V I H R P H K T N I E V S A L P I A K F D R F G V Q V H R
tr:Q8I0K6:Q8I0K6_DROME	R M T I T D H M P L V I H R P H K T N I E V S A L P I A K F D R F G V Q V H R
tr:Q8I0A1:Q8I0A1_DROME	R M T I T D H M P L V I H R P H K T N I E V S A L P I A K F D R F G V Q V H R
tr:Q8SXT8:Q8SXT8_DROME	R M T I T D H M P L V I H R P H K T N I E V S A L P I A K F D R F G V Q V H R
tr:Q8I0B2:Q8I0B2_DROME	R M T I T D H M P L V I H R P H K T N I E V S A L P I A K F D R F G V Q V H R
tr:Q7JQX9:Q7JQX9_DROME	R M T I T D H M P L V I H R P H K T N I E V S A L P I A K F D R F G V Q V H R
tr:Q9VDD2:Q9VDD2_DROME	R M T I T D H M P L V I H R P H K T N I E V S A L P I A K F D R F G V Q V H R
tr:Q9BHL6:Q9BHL6_CAEEL	R M T I T D D R P F V V H R P H K A D I L V S A L P I A K F D R F G V Q V H R
sp:P12904:AAKG_YEAST	V L T T T D D L D T A S G R P Q Y T N M K V S S V P V E A Y D R F G V Y V H R
tr:F4ZZV2:F4ZZV2_YEASX	V L T T T D D L D T A S G R P Q Y T N M K V S S V P V E A Y D R F G V Y V H R

protein	whole protein ident	simil	domain-based ident	simil	contact-based ident	simil
PDB:2v92:chainE:P80385	1.0	1.0	1.0	1.0	1.0	1.0
PDB:2v9j:chainE:P80385	1.0	1.0	1.0	1.0	1.0	1.0
PDB:4eag:chainC:P80385	1.0	1.0	1.0	1.0	0.98	0.95
PDB:4eaj:chainC:P80385	1.0	1.0	1.0	1.0	0.98	0.95
PDB:4eak:chainC:P80385	1.0	1.0	1.0	1.0	0.95	0.91
tr:F8VYY9:F8VYY9_HUMAN	0.83	0.84	0.86	0.86	1.0	1.0
sp:P54619:AAKG1_HUMAN	0.96	0.99	0.98	1.0	1.0	1.0
sp:P80385:AAKG1_RAT	1.0	1.0	1.0	1.0	1.0	1.0
sp:O54950:AAKG1_MOUSE	0.98	0.99	0.98	0.99	1.0	1.0
tr:Q3TWR3:Q3TWR3_MOUSE	0.98	0.99	0.98	0.99	1.0	1.0
tr:Q7KNS9:Q7KNS9_DROME	0.39	0.54	0.64	0.87	0.89	0.91
tr:Q8IGX0:Q8IGX0_DROME	0.36	0.53	0.63	0.88	0.89	0.91
tr:Q0KI34:Q0KI34_DROME	0.37	0.53	0.63	0.88	0.89	0.91
tr:A1A703:A1A703_DROME	0.32	0.46	0.64	0.88	0.89	0.91
tr:A1A6Y3:A1A6Y3_DROME	0.32	0.46	0.64	0.88	0.89	0.91
tr:O96613:O96613_DROME	0.31	0.44	0.64	0.88	0.89	0.91
tr:Q8I0K6:Q8I0K6_DROME	0.28	0.4	0.64	0.88	0.89	0.91
tr:Q8I0A1:Q8I0A1_DROME	0.24	0.35	0.64	0.88	0.89	0.91
tr:Q8SXT8:Q8SXT8_DROME	0.22	0.31	0.64	0.88	0.89	0.91
tr:Q8I0B2:Q8I0B2_DROME	0.22	0.31	0.64	0.88	0.89	0.91
tr:Q7JQX9:Q7JQX9_DROME	0.21	0.3	0.64	0.88	0.89	0.91
tr:Q9VDD2:Q9VDD2_DROME	0.14	0.2	0.64	0.88	0.89	0.91
tr:Q9BHL6:Q9BHL6_CAEEL	0.29	0.47	0.54	0.83	0.77	0.88
sp:P12904:AAKG_YEAST	0.29	0.67	0.33	0.75	0.48	0.67
tr:F4ZZV2:F4ZZV2_YEASX	0.29	0.67	0.33	0.75	0.48	0.67

SNF4Agamma (FBgn0264357) associated phenotypes
feeding behavior defective, hyperactive, neuroanatomy defective, nutrition conditional, phototaxis defective, short lived, starvation stress response defective

(Information from FlyBase)

Dual specificity mitogen-activated protein kinase kinase 1

Best gene implication in ageing for this target family came from gene Q02750 annotated in UniProt release 2014_02. Annotation GO subterm of 7568 (aging) was Inferred from Mutant Phenotype

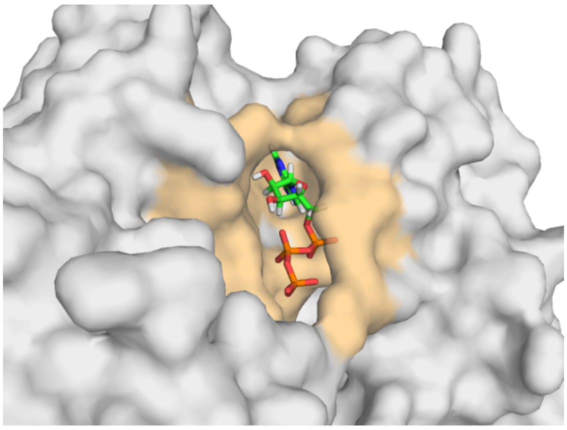

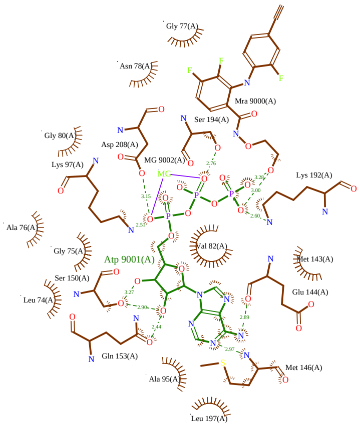

protein	amino acids contacts (binding site)
PDB:1s9j:chainA:Q02750	L G A G N G V A K M E H M S D Q K S N L D
PDB:2p55:chainA:Q02750	L G A G N G V A K M E H M S D Q K S N L D
PDB:3dv3:chainA:Q02750	L G A G N G V A K M E H M S D Q K S N L D
PDB:3dy7:chainA:Q02750	L G A G N G V A K M E H M S D Q K S N L D
PDB:3e8n:chainA:Q02750	L G A G N G V A K M E H M S D Q K S N L D
PDB:3eqb:chainA:Q02750	L G A G N G V A K M E H M S D Q K S N L D
PDB:3os3:chainA:Q02750	L G A G N G V A K M E H M S D Q K S N L D
PDB:3pp1:chainA:Q02750	L G A G N G V A K M E H M S D Q K S N L D
PDB:3v01:chainA:Q02750	L G A G N G V A K M E H M S D Q K S N L D
PDB:3v04:chainA:Q02750	L G A G N G V A K M E H M S D Q K S N L D
PDB:3vvh:chainA:Q02750	L G A G N G V A K M E H M S D Q K S N L D
PDB:4an3:chainA:Q02750	L G A G N G V A K M E H M S D Q K S N L D
sp:Q02750:MP2K1_HUMAN	L G A G N G V A K M E H M S D Q K S N L D
tr:A4QPA9:A4QPA9_HUMAN	L G A G N G V A K M E H M S D Q K S N L D
sp:Q01986:MP2K1_RAT	L G A G N G V A K M E H M S D Q K S N L D
tr:Q3TMJ8:Q3TMJ8_MOUSE	L G A G N G V A K M E H M S D Q K S N L D
sp:P31938:MP2K1_MOUSE	L G A G N G V A K M E H M S D Q K S N L D
sp:Q24324:DSOR1_DROME	L G S G N G V A K M E Y M S D L K S N L D
sp:Q10664:MEK2_CAEEL	L G H G N G V A K M E Y M S D I K S N L D
sp:P08018:PBS2_YEAST	L G H G N G V A K M E Y M S D K K T N L D

	whole protein		domain-based		contact-based	
protein	ident	simil	ident	simil	ident	simil
PDB:1s9j:chainA:Q02750	1.0	1.0	1.0	1.0	1.0	1.0
PDB:2p55:chainA:Q02750	1.0	1.0	1.0	1.0	1.0	1.0
PDB:3dv3:chainA:Q02750	1.0	1.0	1.0	1.0	1.0	1.0
PDB:3dy7:chainA:Q02750	1.0	1.0	1.0	1.0	1.0	1.0
PDB:3e8n:chainA:Q02750	1.0	1.0	1.0	1.0	1.0	1.0
PDB:3eqb:chainA:Q02750	1.0	1.0	1.0	1.0	1.0	1.0
PDB:3os3:chainA:Q02750	1.0	1.0	1.0	1.0	1.0	1.0
PDB:3pp1:chainA:Q02750	1.0	1.0	1.0	1.0	1.0	1.0
PDB:3v01:chainA:Q02750	1.0	1.0	1.0	1.0	1.0	1.0
PDB:3v04:chainA:Q02750	1.0	1.0	1.0	1.0	1.0	1.0
PDB:3vvh:chainA:Q02750	1.0	1.0	1.0	1.0	1.0	1.0
PDB:4an3:chainA:Q02750	0.98	0.99	0.98	0.99	1.0	1.0
sp:Q02750:MP2K1_HUMAN	1.0	1.0	1.0	1.0	1.0	1.0
tr:A4QPA9:A4QPA9_HUMAN	1.0	1.0	1.0	1.0	1.0	1.0
sp:Q01986:MP2K1_RAT	0.99	1.0	1.0	1.0	1.0	1.0
tr:Q3TMJ8:Q3TMJ8_MOUSE	0.99	1.0	1.0	1.0	1.0	1.0
sp:P31938:MP2K1_MOUSE	0.99	1.0	1.0	1.0	1.0	1.0
sp:Q24324:DSOR1_DROME	0.6	0.82	0.75	0.93	0.86	0.86
sp:Q10664:MEK2_CAEEL	0.47	0.74	0.62	0.88	0.86	0.87
sp:P08018:PBS2_YEAST	0.2	0.36	0.45	0.74	0.81	0.87

Dsor1 (FBgn0010269) associated phenotypes

dominant, lethal - all die before end of pupal stage, neuroanatomy defective, polyphasic, some die during immature adult stage, some die during pupal stage

(Information from FlyBase)

Dsor1 (UniProt:Q24324) annotation

Function: Required downstream of Raf in the sevenless (sev), torso (tor), and Drosophila EGF receptor homolog (DER) signal transduction pathways. Involved in both positive regulation (at the posterior terminus) and negative regulation (at the anterior domain) of tll, as in other terminal class gene products, maybe via the ERK-A kinase. (PubMed:8381718).
Developmental stage: Expressed both maternally and zygotically. (PubMed:8381718).
Ptm: Phosphorylation on Ser/Thr by MAP kinase kinase kinases regulates positively the kinase activity.

(Information from UniProt)

mek-2 (WBGene00003186) associated phenotypes

AWA odorant chemotaxis defective, AWC odorant chemotaxis defective, bacterially unswollen, constipated, germ cell arrest, germ nuclei rachis, lethal, metabolic pathway variant, no oocytes, pachytene region organization variant, pathogen susceptibility increased, rod like larval lethal, sterile, vulval cell lineage variant, vulvaless

(Information from WormBase)

mek-2 (UniProt:Q10664) annotation

Function: Functions in the let-60 Ras signaling pathway; acts downstream of lin-45 raf kinase, but before the sur-1/mpK-1 gene product in controlling vulval cell differentiation.
Enzyme regulation: Activated by tyrosine and threoninephosphorylation catalyzed by MAP kinase kinase kinases.

(Information from UniProt)

Focal adhesion kinase 1

Best gene implication in ageing for this target family came from gene Q05397 via mapping the annotation from Ensembl ENSG00000169398 via mapping the annotation from EntrezGene 5747 via mapping the annotation from GenAgeHuman 0166 annotated in GenAge release 17.

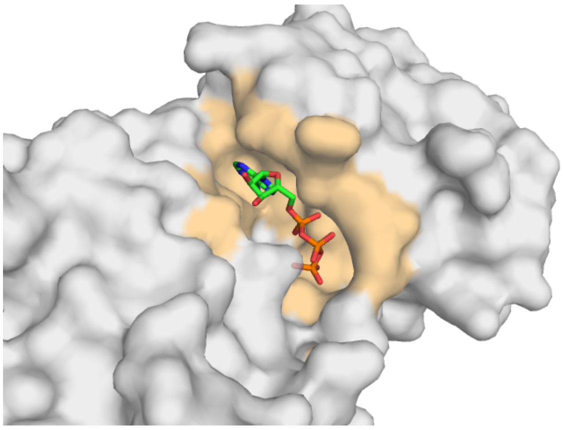

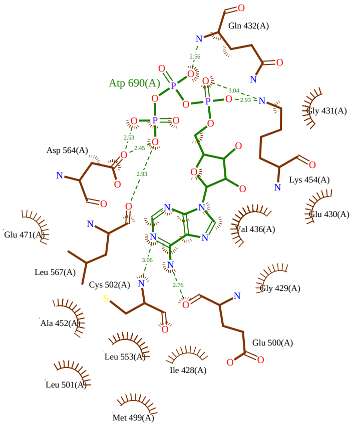

protein	amino acids contacts (binding site)
PDB:2ijm:chainA:Q05397	I G E G Q V A K E M E L C E N L D F L
tr:E9PEI4:E9PEI4_HUMAN	I G E G Q V A K E M E L C E N L D F L
tr:H0YB16:H0YB16_HUMAN	I G E G Q V A K E M E L C E N L D F L
tr:Q8N9D7:Q8N9D7_HUMAN	I G E G Q V A K E M E L C E N L D F L
tr:B4DH13:B4DH13_HUMAN	I G E G Q V A K E M E L C E N L D F L
tr:H0YBZ1:H0YBZ1_HUMAN	I G E G Q V A K E M E L C E N L D F L
tr:Q8IYN9:Q8IYN9_HUMAN	I G E G Q V A K E M E L C E N L D F L
sp:Q05397:FAK1_HUMAN	I G E G Q V A K E M E L C E N L D F L
tr:J3QT16:J3QT16_HUMAN	I G E G Q V A K E M E L C E N L D F L
tr:E7ESA6:E7ESA6_HUMAN	I G E G Q V A K E M E L C E N L D F L
tr:R9PXT6:R9PXT6_RAT	I G E G Q V A K E M E L C E N L D F L
sp:P34152:FAK1_MOUSE	I G E G Q V A K E M E L C E N L D F L
tr:Q2TGI0:Q2TGI0_DROME	I G V G Q V A K E M E L A E N L D F L
tr:Q9U5Y2:Q9U5Y2_DROME	I G V G Q V A K E M E L A E N L D F L
tr:E1JGM8:E1JGM8_DROME	I G V G Q V A K E M E L A E N L D F L
tr:B8A418:B8A418_DROME	I G V G Q V A K E M E L A E N L D F L
tr:Q5BIG9:Q5BIG9_DROME	I G V G Q V A K E M E L A E N L D F L
tr:Q0E917:Q0E917_DROME	I G V G Q V A K E M E L A E N L D F L
tr:Q9U531:Q9U531_DROME	I G V G Q V A K E M E L A E N L D F L
tr:Q9U472:Q9U472_DROME	I G V G Q V A K E M E L A E N L D F L
tr:B7YZL9:B7YZL9_DROME	I G V G Q V A K E M E L A E N L D F L
tr:Q8T879:Q8T879_CAEEL	I G G G Q V A K E L E L A E N L D F L
tr:Q95YD4:Q95YD4_CAEEL	I G G G Q V A K E L E L A E N L D F L

	whole protein		domain-based		contact-based	
protein	ident	simil	ident	simil	ident	simil
PDB:2ijm:chainA:Q05397	1.0	1.0	1.0	1.0	1.0	1.0
tr:E9PEI4:E9PEI4_HUMAN	0.63	0.64	1.0	1.0	1.0	1.0
tr:H0YB16:H0YB16_HUMAN	0.68	0.68	0.97	0.97	1.0	1.0
tr:Q8N9D7:Q8N9D7_HUMAN	0.67	0.67	0.97	0.97	1.0	1.0
tr:B4DH13:B4DH13_HUMAN	0.65	0.65	0.98	0.98	1.0	1.0
tr:H0YBZ1:H0YBZ1_HUMAN	0.66	0.66	0.98	0.98	1.0	1.0
tr:Q8IYN9:Q8IYN9_HUMAN	0.96	0.96	1.0	1.0	1.0	1.0
sp:Q05397:FAK1_HUMAN	1.0	1.0	1.0	1.0	1.0	1.0
tr:J3QT16:J3QT16_HUMAN	0.99	0.99	1.0	1.0	1.0	1.0
tr:E7ESA6:E7ESA6_HUMAN	0.96	0.96	1.0	1.0	1.0	1.0
tr:R9PXT6:R9PXT6_RAT	0.97	0.99	0.99	1.0	1.0	1.0
sp:P34152:FAK1_MOUSE	0.94	0.96	0.95	0.95	1.0	1.0
tr:Q2TGI0:Q2TGI0_DROME	0.16	0.23	0.56	0.79	0.89	0.84
tr:Q9U5Y2:Q9U5Y2_DROME	0.29	0.61	0.56	0.79	0.89	0.84
tr:E1JGM8:E1JGM8_DROME	0.29	0.62	0.56	0.79	0.89	0.84
tr:B8A418:B8A418_DROME	0.29	0.62	0.56	0.79	0.89	0.84
tr:Q5BIG9:Q5BIG9_DROME	0.29	0.62	0.56	0.79	0.89	0.84
tr:Q0E917:Q0E917_DROME	0.29	0.62	0.56	0.79	0.89	0.84
tr:Q9U531:Q9U531_DROME	0.29	0.62	0.56	0.79	0.89	0.84
tr:Q9U472:Q9U472_DROME	0.29	0.62	0.56	0.79	0.89	0.84
tr:B7YZL9:B7YZL9_DROME	0.24	0.52	0.56	0.79	0.89	0.84
tr:Q8T879:Q8T879_CAEEL	0.21	0.54	0.47	0.77	0.84	0.84
tr:Q95YD4:Q95YD4_CAEEL	0.21	0.57	0.47	0.77	0.84	0.84

Fak (FBgn0020440) associated phenotypes

neuroanatomy defective, neurophysiology defective

(Information from FlyBase)

Integrin-linked protein kinase

Best gene implication in ageing for this target family came from gene Q99J82 annotated in UniProt release 2014_02. Annotation GO subterm of 7568 (aging) was Inferred from Mutant Phenotype

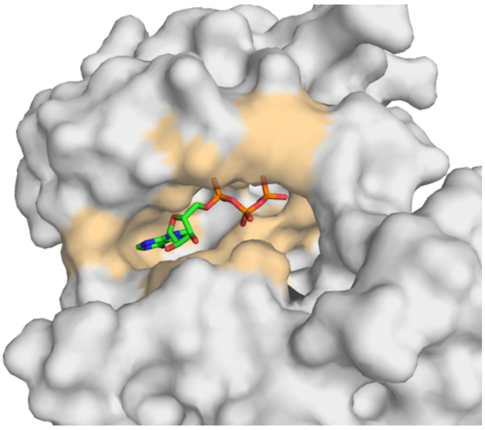

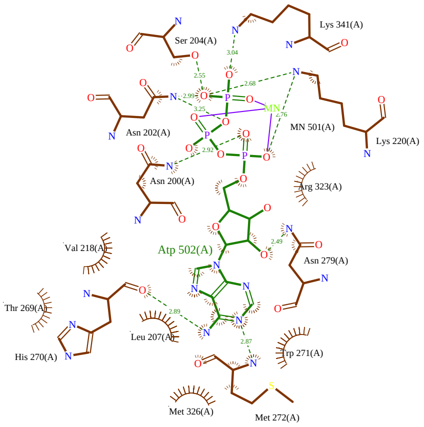

protein	amino acids contacts (binding site)															
PDB:3kmw:chainA:Q13418	N	N	H	S	L	V	K	T	H	W	M	N	R	M	D	K
PDB:3rep:chainA:Q13418	N	N	H	S	L	V	K	T	H	W	M	N	R	M	D	K
tr:B7Z1I0:B7Z1I0_HUMAN	N	N	H	S	L	V	K	T	H	W	M	N	R	M	D	K
sp:Q13418:ILK_HUMAN	N	N	H	S	L	V	K	T	H	W	M	N	R	M	D	K
sp:Q99J82:ILK_RAT	N	N	H	S	L	V	K	T	H	W	M	N	R	M	D	K
sp:O55222:ILK_MOUSE	N	N	H	S	L	V	K	T	H	W	M	N	R	M	D	K
tr:Q9NHC7:Q9NHC7_DROME	S	T	P	S	T	V	K	S	Q	F	M	S	H	M	D	K
tr:Q9V400:Q9V400_DROME	S	T	P	S	T	V	K	S	Q	F	M	S	H	M	D	K
tr:Q9NG00:Q9NG00_CAEEL	A	S	H	S	L	V	R	S	Q	Y	M	N	K	V	D	K
tr:Q9TZC4:Q9TZC4_CAEEL	A	S	H	S	L	V	R	S	Q	Y	M	N	K	V	D	K

	whole protein		domain-based		contact-based	
protein	ident	simil	ident	simil	ident	simil
PDB:3kmw:chainA:Q13418	0.99	1.0	0.99	1.0	1.0	1.0
PDB:3rep:chainA:Q13418	0.99	1.0	0.99	1.0	1.0	1.0
tr:B7Z1I0:B7Z1I0_HUMAN	0.7	0.7	1.0	1.0	1.0	1.0
sp:Q13418:ILK_HUMAN	1.0	1.0	1.0	1.0	1.0	1.0
sp:Q99J82:ILK_RAT	1.0	1.0	0.99	1.0	1.0	1.0
sp:O55222:ILK_MOUSE	1.0	1.0	0.99	1.0	1.0	1.0
tr:Q9NHC7:Q9NHC7_DROME	0.59	0.86	0.6	0.87	0.44	0.74
tr:Q9V400:Q9V400_DROME	0.6	0.86	0.62	0.87	0.44	0.74
tr:Q9NG00:Q9NG00_CAEEL	0.58	0.86	0.58	0.87	0.5	0.8
tr:Q9TZC4:Q9TZC4_CAEEL	0.56	0.83	0.58	0.87	0.5	0.8

Ilk (FBgn0028427) associated phenotypes

lethal - all die before end of first instar larval stage, somatic clone

(Information from FlyBase)

pat-4 (WBGene00003931) associated phenotypes

body wall muscle actin organization defective, body wall muscle myosin organization defective, paralyzed arrested elongation two fold

(Information from WormBase)
